# Repurposing amlodipine, fluoxetine, and escitalopram as non-antibiotic adjuvants: in vitro enhancement of ciprofloxacin and fluconazole activity against multidrug-resistant bacterial and *candida* clinical isolates

**DOI:** 10.1186/s12866-026-05443-4

**Published:** 2026-07-23

**Authors:** Mohammed A. Alkhawlani, Wafa M. Al-Madhagi, Mahmoud Mahyoob Alburyhi, Ali Gamal Al-kaf, Maged Al-Najar

**Affiliations:** 1 Department of Pharmacology, Faculty of Pharmacy, Sana’a University, Sana’a, Yemen; 2 Department of Pharmaceutical Chemistry, Faculty of Pharmacy, Sana’a University, Sana’a, Yemen; 3 Department of Pharmaceutics and Industrial Pharmacy, Faculty of Pharmacy, Sana’a University, Sana’a, Yemen; 4 Department of Medicinal Chemistry, Faculty of Pharmacy, Sana’a University, Sana’a, Yemen; 5 Department of Clinical Pharmacy and Pharmacy Practice, Faculty of Pharmacy, University of Malaya, Kuala Lumpur, Malaysia

**Keywords:** Amlodipine, Escitalopram, Fluoxetine, Antimicrobial resistance, Drug repurposing, Antimicrobial adjuvant

## Abstract

**Introduction:**

Multidrug-resistant (MDR) *Staphylococcus aureus*, *Pseudomonas aeruginosa*, and *Candida albicans* threaten treatment success, particularly in resource-limited settings. Repurposing licensed, inexpensive non-antibiotic drugs may provide a strategy for enhancing the activity of established antimicrobial agents.

**Methods:**

Clinical isolates from Sana’a, Yemen, and American Type Culture Collection (ATCC) reference strains were tested in vitro with amlodipine, fluoxetine, and escitalopram, individually and in dual or triple combinations with ciprofloxacin or fluconazole. Antimicrobial activity was evaluated using agar-well diffusion, broth microdilution minimum inhibitory concentration (MIC), minimum bactericidal or fungicidal concentration (MBC/MFC), and checkerboard fractional inhibitory concentration index (FICI) assays. Molecular docking with AutoDock Vina was used to explore plausible interactions with MurB (PDB 1HSK), LasR (PDB 3IX3), and CYP51 (PDB 5V5Z). Normal mode analysis (NMA) with iMODS was performed for selected fluoxetine–LasR and fluoxetine–MurB complexes as a preliminary structural-dynamics assessment.

**Results:**

Fluoxetine produced inhibition zones of at least 27 mm across the tested organisms. Amlodipine produced larger zones against *S. aureus* and *C. albicans* than against *P. aeruginosa*, whereas escitalopram produced smaller zones overall. Checkerboard testing identified synergy (FICI ≤ 0.50) for selected combinations. Selected triple combinations reduced MIC values by 4- to 64-fold. The largest diffusion readouts were observed with ciprofloxacin + fluoxetine + amlodipine against *S. aureus* (101 mm), ciprofloxacin + fluoxetine + escitalopram against *P. aeruginosa* (88.5 mm), and ciprofloxacin + amlodipine + fluconazole against *C. albicans* (138.5 mm). Docking scores and NMA outputs provided hypothesis-generating structural context for the experimental findings.

**Conclusions:**

Antimicrobial enhancement was combination- and organism-dependent. Selected combinations showed checkerboard-defined synergy, whereas diffusion assays were interpreted as screening outcomes only. Docking and NMA findings were supportive and hypothesis-generating but did not establish target engagement or mechanism.

**Supplementary Information:**

The online version contains supplementary material available at https://doi.org/10.1186/s12866-026-05443-4.

## Introduction

Antimicrobial resistance (AMR) is widely recognized as one of the gravest threats to global public health, jeopardizing decades of progress in infection control and patient safety. In 2019 alone, bacterial AMR was associated with an estimated 4.95 million deaths, of which 1.27 million were directly attributable to drug-resistant infections [1]. A recent global analysis forecasts that the burden of bacterial AMR will remain substantial through 2050 [2]. Beyond mortality, AMR increases the likelihood of treatment failure and complications, prolongs illness, and is associated with longer hospital stays and the need for more expensive alternative therapies, thereby increasing the economic burden on both health systems and patients [3, 4]. AMR also undermines the safety of many routine and life-saving medical interventions (e.g., major surgery, caesarean section, organ transplantation, and cancer chemotherapy) in which effective antimicrobial prophylaxis and treatment are essential [5, 6]. Collectively, these clinical and economic harms highlight the urgency of identifying adjunct strategies—such as drug repurposing—to restore or potentiate the activity of existing antimicrobials [7, 8]. In parallel with diminishing treatment options, the clinical pipeline for novel antimicrobials remains alarmingly thin [9]. These trends underscore the urgent need to explore innovative strategies—including the repurposing of licensed, non-antibiotic drugs—for their potential antibacterial and antifungal activities.

Amlodipine, fluoxetine, and escitalopram represent three such candidates. Amlodipine is a dihydropyridine calcium-channel blocker indicated for hypertension and angina, and experimental studies have reported antimicrobial activity for amlodipine in vitro and in infection models [10]. Fluoxetine and escitalopram, selective serotonin reuptake inhibitors (SSRIs) widely prescribed for major depressive and anxiety disorders, also display direct antimicrobial effects—ranging from bactericidal activity against *Staphylococcus aureus* to growth inhibition of *Candida albicans* [11, 12]. Although the concentrations required for antimicrobial action exceed typical plasma levels, these non-antibiotic agents can act as pharmacological adjuvants that sensitize microbes to conventional drugs or impair resistance mechanisms such as efflux pump activity [13].

Combining repurposed medications with frontline agents may therefore enhance antimicrobial activity against recalcitrant pathogens. Ciprofloxacin, a fluoroquinolone antibiotic, and fluconazole, an azole antifungal, remain cornerstones of treatment for bacterial and fungal infections, respectively; however, resistance among *S. aureus*, *Pseudomonas aeruginosa*, and *C. albicans* is increasingly reported [14]. Experimental studies have reported fluoxetine–ciprofloxacin potentiation [13] and fluconazole synergy with calcium-channel blockers against resistant *C. albicans* [15].

We selected amlodipine, fluoxetine, and escitalopram as repurposing candidates based on (i) published evidence that some non-antibiotic cardiovascular and psychotropic agents can exhibit intrinsic antimicrobial activity and/or antibiotic-adjuvant effects, (ii) wide availability and low cost in many low-resource settings, and (iii) mechanistic plausibility for sensitizing multidrug-resistant (MDR) pathogens by altering permeability, inhibiting efflux, or attenuating biofilm-associated phenotypes. Amlodipine (a dihydropyridine calcium-channel blocker) has been described as an antimicrobial “helper compound” with in vitro synergy against bacterial pathogens in prior work [16]. Selective serotonin reuptake inhibitors (SSRIs) such as fluoxetine have shown antibacterial and antibiofilm activity and have been reported to inhibit bacterial efflux in experimental systems [17].

These observations motivated us to test whether pairing these repurposed agents with first-line anchors could restore susceptibility in local clinical isolates. To generate mechanistic hypotheses consistent with the organisms and anchors tested, we focused in silico analyses on three well-characterized targets representing distinct, clinically relevant pathways: UDP-N-acetylenolpyruvylglucosamine reductase (MurB) for bacterial peptidoglycan biosynthesis [18], LasR as a quorum-sensing transcriptional regulator implicated in virulence and biofilm-associated tolerance in *Pseudomonas aeruginosa* [19], and fungal lanosterol 14α-demethylase (CYP51) as the canonical azole target in the ergosterol pathway [20]. Docking was used to explore whether the repurposed drugs could plausibly interact with these targets in ways consistent with the observed in vitro interaction patterns.

The present study aimed to evaluate the antibacterial and antifungal activities of amlodipine, fluoxetine, and escitalopram against clinical isolates of *S. aureus*, *P. aeruginosa*, and *C. albicans*, both alone and in combination with ciprofloxacin or fluconazole.

## Materials and methods

### Chemicals, reagents, and instrumentation

#### Drugs and Chemicals

Amlodipine 20 mg, ciprofloxacin 250 mg, escitalopram 40 mg, and fluconazole 200 mg were supplied by Yemen Egyptian Pharmaceutical Industry; fluoxetine 40 mg was supplied by Rafa Pharmaceutical Industry. Powdered blood agar, brain–heart infusion broth, cystine–lactose–electrolyte-deficient (CLED) agar, and Mueller–Hinton agar (Oxoid, UK) was prepared according to the manufacturer’s instructions. All other analytical-grade reagents were used as received; the supplier list is provided in Table S1.

#### Instrumentation

Glassware and media were sterilized in a steam autoclave (SX-700, Tomy, Japan; 121 °C, 15 psi, 15 min). Cultures were incubated at 30–37 °C in a forced-air incubator (IN110, Memmert, Germany). Reference strains were reauthenticated using a VITEK 2 COMPACT analyzer (bioMérieux, France). Optical density and endpoint absorbance at 405, 450, and 620 nm for MIC, MBC/MFC, and FIC assays were recorded using a Multiskan EX microplate reader (Thermo Scientific, USA). Liquid handling was performed with calibrated Research plus pipettes (Eppendorf, Germany). Detailed equipment specifications are provided in Table S2.

### Microorganisms

#### Bacterial Strains

Reference strains *S. aureus* ATCC 29,213 and *P. aeruginosa* ATCC 27,853 were obtained from YEDCO for Pharmaceutical Industries. Clinical bacterial isolates were recovered from pus swab specimens submitted to the NCPHL in Sana’a, Yemen. The *C. albicans* clinical isolate used in this study was obtained from the NCPHL collection of isolates recovered from vaginal swab, oral swab, and urine specimens.

#### Fungal Strain

Clinical *C. albicans* isolates were identified using standard microbiological and biochemical methods; the isolate included in antimicrobial testing was selected from the confirmed NCPHL collection.

#### Quality-control reference strains

To ensure the validity of susceptibility testing, certified American Type Culture Collection (ATCC) strains were tested alongside the clinical isolates. *S. aureus* ATCC 25,923 was used as the disk-diffusion quality-control strain, whereas *S. aureus* ATCC 29,213, *P. aeruginosa* ATCC 27,853, and *C. albicans* ATCC 90,028 served as reference strains for broth microdilution and checkerboard assays. Lyophilized cultures were reconstituted according to the supplier’s instructions, subcultured twice on tryptic soy agar (bacteria) or Sabouraud dextrose agar (yeast), and adjusted to a 0.5 McFarland standard before each run. Zone diameters and MIC values for the ATCC strains were within the specified quality-control ranges, supporting the validity of the test conditions [21].

### Study area and period

All microbial isolation, identification, and antimicrobial susceptibility tests were conducted at the Microbiology Department of the National Center of Public Health Laboratories (NCPHL), Ministry of Health and Population, Sana’a, Yemen. The study was conducted from 1 October 2023 to 1 February 2024.

### Experimental procedures

All experiments were performed under aseptic conditions using the microbiological procedures described below.

#### Isolation, identification, and sub-culturing of microorganisms

For the reference strains, the identity and purity of *S. aureus* ATCC 29,213 and *P. aeruginosa* ATCC 27,853 were reconfirmed using a VITEK^®^ 2 COMPACT analyzer (bioMérieux, France) with ID-GP and ID-GN cards, respectively. Both strains showed at least 95% probability for the expected species, and internal quality-control values for ciprofloxacin and gentamicin were within the acceptance limits specified in the VITEK^®^ 2 AST card package insert.

For clinical isolates, pus samples were collected in sterile containers and transported to the laboratory within six hours. Identification of these isolates was performed based on colony morphology and a series of biochemical tests.

#### Microscopic and biochemical identification

Gram staining, catalase testing, DNase testing for *S. aureus*, oxidase testing for *P. aeruginosa*, and carbohydrate-assimilation testing for *C. albicans* were performed using standard protocols; positive controls were included in every batch.

### Antimicrobial activity

#### Media preparation

Mueller–Hinton agar (38 g L⁻¹) and cation-adjusted Mueller–Hinton broth (CAMHB; Ca²⁺ 25 mg L⁻¹, Mg²⁺ 12.5 mg L⁻¹) were prepared and quality-controlled with *E. coli* ATCC 25,922 before use. Columbia blood agar (Oxoid, UK) was prepared by autoclaving the base medium (40.5 g L⁻¹) at 121 °C for 15 min, cooling it to 50 °C, and aseptically adding 5% (v/v) defibrinated sheep blood. Plates with an external diameter of 150 mm and an internal diameter of approximately 140 mm (Cat. No. P605, Polylab^®^, India) were poured, allowed to solidify, and stored at 4 °C for no longer than 1 week [21]. Only cultures that passed quality-control checks and showed a single expected hemolysis pattern were advanced to susceptibility testing.

#### Drug-solution preparation

Stock solutions were prepared separately for diffusion and broth-based assays using the assay-specific solvents described below. Working solutions were prepared immediately before testing, and solvent concentrations were matched across single-agent, combination, and vehicle-control conditions.

##### Agar-well diffusion (ethanol stocks)

For diffusion assays, compounds were first dissolved in absolute ethanol. Immediately before use, the stocks were diluted with sterile distilled water to prepare working solutions of 1 mg mL⁻¹ for ciprofloxacin and fluconazole and 2 mg mL⁻¹ for amlodipine, fluoxetine, and escitalopram. The final ethanol concentration was ≤ 2% (v/v) in all wells and was solvent-matched across single-drug and combination arms. Each plate included vehicle-control wells containing the corresponding ethanol–water solution (≤ 2% v/v) without drug.

##### Broth MIC/MBC/MFC/ΣFIC (DMSO stocks)

For broth microdilution and checkerboard assays, concentrated stocks were prepared in DMSO and diluted into cation-adjusted Mueller–Hinton broth (CAMHB) such that the final DMSO content in all wells (drug, combination, and controls) was ≤ 1% (v/v). DMSO-only vehicle controls were included on every plate and showed no growth inhibition relative to growth controls.

#### Agar well diffusion assay

Assays were performed in sterile 150-mm Petri dishes containing approximately 25 mL of Mueller–Hinton agar at a depth of approximately 4 mm. After solidification, 5-mm wells were bored at least 10 mm from the plate edge and at least 15 mm apart. Each well received 100 µL of vehicle control (≤ 2% ethanol in water), a single-agent working solution, or a premixed combination. The final ethanol concentration was ≤ 2% (v/v) and was identical across the corresponding single-agent and combination conditions.

#### Combination antimicrobial testing

Dual and triple combinations were evaluated using the agar-well diffusion method. Because standardized diffusion-based criteria are not available for classifying drug interactions, a conservative Δ-zone approach was used for descriptive interpretation by comparing each combination zone with the largest corresponding single-agent zone on the same plate. Increases of ≥ 5 mm was classified as marked zone enhancement, increases of 2–4 mm as modest zone enhancement, changes of ≤ 1 mm as no material change, and reductions of ≥ 2 mm as reduced inhibition. These categories were used for qualitative screening only and were not used to define synergy [22, 23]. Synergy was defined as a priori using the broth checkerboard fractional inhibitory concentration index (ΣFIC ≤ 0.50) for all organisms [24, 25]. Accordingly, all synergy claims were based on checkerboard results, whereas diffusion readouts were treated as screening outcomes.

#### Minimum inhibitory concentration (MIC)

MICs were determined by broth microdilution following Wiegand et al. [26] using CAMHB. Briefly, 100 µL of CAMHB was dispensed into each well of sterile 96-well microplates. Drug stocks prepared in DMSO were added to the first column, and twofold serial dilutions were performed across the plate. The final DMSO concentration was ≤ 1% (v/v) and solvent-matched in all wells. A 5-µL inoculum adjusted to a 0.5 McFarland standard (approximately 1–2 × 10⁸ CFU mL⁻¹; final well inoculum, 5–10 × 10⁵ CFU mL⁻¹) was added to each well. Every plate contained medium control without inoculum, a growth control containing inoculum without DMSO, and a vehicle control containing inoculum plus DMSO at the same concentration used in the drug wells. Plates were incubated at 37 °C for 18–24 h. Absorbance at 620 nm was used as the primary turbidity measure. Reads at 405 and 450 nm were used only to verify borderline trailing growth or optical artifacts. MICs were determined relative to solvent-matched vehicle controls. Fluconazole was included only as the antifungal anchor for *Candida* assays. Fluconazole-containing wells on bacterial plates were internal negative or specificity controls and were not used for antibacterial interaction classification. Each condition was tested in duplicate (*n* = 2).

#### Minimum bactericidal or fungicidal concentration (MBC/MFC)

From wells at or above the MIC, 10-µL aliquots were spot-plated onto the corresponding drug-free agar and incubated at 37 °C for 24 h. The minimum bactericidal concentration (MBC) for bacterial strains or minimum fungicidal concentration (MFC) for *C. albicans* was defined as the lowest concentration yielding no colony growth. Each condition was tested in duplicate (*n* = 2).

#### Checkerboard assay and FICI calculation

Checkerboard assays were used to evaluate interactions between agents from different drug classes. All assays were conducted under solvent-matched conditions, with DMSO maintained at ≤ 1% (v/v) in all wells, including vehicle controls [23]. For each agent, the fractional inhibitory concentration (FIC) was calculated by dividing its MIC in combination by its MIC alone.

$$\mathrm{FIC}_\mathrm{a} = \text{MIC of drug A in combination / MIC of drug A alone.}$$

For three-drug combinations, the fractional inhibitory concentration index was calculated as ΣFIC = FICₐ + FIC_β_ + FIC𝒸. Interactions were interpreted as synergistic (ΣFIC ≤ 0.5), additive or partially synergistic (0.5 < ΣFIC ≤ 1), indifferent (1 < ΣFIC ≤ 4), or antagonistic (ΣFIC > 4).

### Molecular docking studies

Molecular docking was used as a hypothesis-generating approach to explore plausible ligand–target interactions consistent with the observed in vitro activity patterns. Targets represented bacterial cell-wall biosynthesis (MurB), quorum-sensing regulation associated with biofilm tolerance (LasR), and the established fungal azole target (CYP51). *S. aureus* MurB (PDB 1HSK) catalyzes an essential step in peptidoglycan biosynthesis [18]. *P. aeruginosa* LasR (PDB 3IX3) is a quorum-sensing transcriptional regulator involved in virulence and biofilm-associated phenotypes [19]. *C. albicans* CYP51 (lanosterol 14α-demethylase; PDB 5V5Z) is the established azole target in the ergosterol biosynthesis pathway [20]. Prior in silico studies have used docking and dynamics to investigate quorum-sensing modulators [27], while benchmarking studies emphasize that docking scores should be interpreted as predictions rather than direct binding measurements [28]. Native ligands and crystallographic water molecules were removed using Discovery Studio 2021, and the prepared structures were imported into PyRx 0.8. Amlodipine, fluoxetine, escitalopram, fluconazole, and ciprofloxacin structures were obtained from PubChem, energy-minimized, and saved in PDB format. AutoDock Vina was run with an exhaustiveness of 20. Grid centers were 178.8 × 150.8 × 163.8 Å for 1HSK, 24.4 × 0.3 × 17.6 Å for 3IX3, and 56.6 × 47.6 × 20.9 Å for 5V5Z; the corresponding box sizes were 70 × 50 × 70, 80 × 65 × 96, and 100 × 115 × 128 Å. Predicted docking scores and interaction profiles were exported and visualized in Discovery Studio [29].

### Normal mode analysis (NMA) of selected docked complexes

To provide a preliminary dynamics-based structural assessment of the selected docked complexes, normal mode analysis (NMA) was performed using the iMODS server. The iMODS platform applies internal-coordinate NMA to explore collective motions, deformability, and structural flexibility of macromolecular complexes and is considered a rapid and computationally efficient approach for evaluating the dynamic behavior of protein–ligand systems [30, 31]. Based on the docking results and their relevance to the in vitro antibacterial findings, two fluoxetine-bound complexes were selected for analysis: fluoxetine docked to *Pseudomonas aeruginosa* LasR (PDB: 3IX3) and fluoxetine docked to *Staphylococcus aureus* MurB (PDB: 1HSK). The best docked poses generated from the molecular docking workflow were exported as PDB files and used as input structures for the iMODS server. Prior to analysis, crystallographic water molecules and non-essential heteroatoms were removed from the docked complex structures. NMA was then performed under the default iMODS settings [31].

The iMODS outputs included mobility vectors, deformability profiles, estimated B-factors, eigenvalues, variance, covariance maps, and elastic network models [31]. These outputs were used to compare predicted collective motions and relative flexibility between the selected protein–fluoxetine complexes. NMA was used only as a preliminary structural-dynamics assessment and was not interpreted as a full molecular dynamics simulation.

## Results

### Identification of microorganisms

Gram staining and biochemical tests were used to identify the clinical bacterial and fungal isolates. Reference strains were confirmed using the VITEK system. All procedures were performed at the National Center of Public Health Laboratories (NCPHL), Sana’a, Yemen. The confirmed isolates and reference strains were then advanced to antimicrobial testing.

### Antimicrobial activity of single agents

In the agar-diffusion screening, fluconazole was included as an antifungal comparator (Table 1). Any limited clearing observed on bacterial plates was not interpreted as antibacterial activity; subsequent interaction claims were based on ciprofloxacin-anchored testing and checkerboard FICI analyses.

**Table 1:** Inhibition-zone diameters (mm) of single agents against clinical isolates and corresponding ATCC reference strains

Drug	Clinical isolates			Reference strains (ATCC)	
	*Staphylococcus aureus* clinical isolate	*Pseudomonas aeruginosa* clinical isolate	*Candida albicans* clinical isolate	*Staphylococcus aureus* ATCC 25,923	*Pseudomonas aeruginosa* ATCC 27,853
Vehicle (≤ 2% EtOH in water)	0.0 ± 0.0	0.0 ± 0.0	0.0 ± 0.0	0.0 ± 0.0	0.0 ± 0.0
Ciprofloxacin	46.0 ± 1.4	39.0 ± 4.2	25.0 ± 0.0	52.0 ± 1.4	62.5 ± 0.7
Fluconazole	19.0 ± 0.0	0.0 ± 0.0	30.0 ± 0.0	23.4 ± 0.6	24.3 ± 0.4
Fluoxetine	40.0 ± 1.4	27.0 ± 1.4	42.0 ± 0.0	43.3 ± 1.0	47.4 ± 0.6
Amlodipine	31.0 ± 1.4	20.0 ± 0.0	41.5 ± 4.9	35.3 ± 0.4	38.2 ± 0.3
Escitalopram	23.5 ± 0.7	15.5 ± 0.7	30.0 ± 0.0	29.7 ± 1.7	39.5 ± 0.7

Values are mean ± sample standard deviation from two independent determinations (*n* = 2). Vehicle = ≤ 2% (v/v) ethanol in water without drug; no measurable clearing occurred beyond the 5-mm well

*ATCC*  American Type Culture Collection, *EtOH*  ethanol. Fluconazole results on bacterial panels were plate controls and were not used for antibacterial inference. Drug-source tablet strengths were amlodipine 20 mg, ciprofloxacin 250 mg, escitalopram 40 mg, fluconazole 200 mg, and fluoxetine 40 mg

Against the clinical bacterial isolates, ciprofloxacin produced the largest inhibition zones in the single-agent diffusion screen: 46.0 ± 1.4 mm against *S. aureus* and 39.0 ± 4.2 mm against *P. aeruginosa*. Among the repurposed agents, fluoxetine produced zones of 40.0 ± 1.4 mm against *S. aureus*, 27.0 ± 1.4 mm against *P. aeruginosa*, and 42.0 ± 0.0 mm against *C. albicans*. Amlodipine produced zones of 31.0 ± 1.4, 20.0 ± 0.0, and 41.5 ± 4.9 mm, respectively, whereas escitalopram produced smaller zones, particularly against *P. aeruginosa* (15.5 ± 0.1 mm). Fluconazole inhibited *C. albicans* (30.0 ± 0.0 mm); limited clearing on bacterial plates was treated as a control observation and was not interpreted as antibacterial activity. In the ATCC reference strains, ciprofloxacin produced zones of 52.0 ± 1.4 mm against *S. aureus* and 62.5 ± 4.2 mm against *P. aeruginosa*. Vehicle controls were non-inhibitory under all tested conditions (Table 1).

### Efficacy of dual combinations

For dual combinations producing very large inhibition zones (≥ 70 mm), assays were conducted on single-well 150-mm plates to prevent overlap and permit accurate measurement; each independent run used a fresh plate. Diffusion assays are reported as screening or phenotypic enhancement readouts, whereas formal synergy classification is based on checkerboard FICI criteria.

#### Dual combinations against the clinical *S. aureus* isolate

Against the *S. aureus* clinical isolate, the largest inhibition zone among dual combinations was observed for fluoxetine + fluconazole (49.0 ± 2.0 mm). High inhibition was also observed for ciprofloxacin + escitalopram (43.5 ± 2.1 mm), ciprofloxacin + fluconazole (41.0 ± 0.0 mm), and fluoxetine + escitalopram (41.0 ± 1.4 mm). Several other dual combinations produced intermediate inhibition (e.g., fluoxetine + amlodipine, 40.5 ± 0.7 mm; fluoxetine + ciprofloxacin, 40.5 ± 1.8 mm). These organism-specific diffusion patterns are summarized in Table 2.

**Table 2:** Inhibition-zone diameters (mm) of dual combinations against clinical isolates of *Staphylococcus aureus*, *Pseudomonas aeruginosa*, and *Candida albicans*

Combination	*Staphylococcus aureus* clinical isolate	*Pseudomonas aeruginosa* clinical isolate	*Candida albicans* clinical isolate
Vehicle (≤ 2% EtOH in water)	0.0 ± 0.0	0.0 ± 0.0	0.0 ± 0.0
Ciprofloxacin + Fluoxetine	40.5 ± 1.8	28.5 ± 1.1	44.0 ± 1.4
Ciprofloxacin + Amlodipine	34.5 ± 0.7	47.5 ± 0.7	33.5 ± 1.8
Ciprofloxacin + Escitalopram	43.5 ± 2.1	46.5 ± 2.1	26.5 ± 0.7
Ciprofloxacin + Fluconazole	41.0 ± 0.0	42.0 ± 0.0	35.5 ± 4.6
Fluconazole + Fluoxetine	49.0 ± 2.0	26.5 ± 0.7	50.5 ± 1.8
Fluconazole + Amlodipine	37.5 ± 3.5	15.0 ± 0.0	45.5 ± 0.7
Fluconazole + Escitalopram	28.0 ± 1.1	16.0 ± 0.0	44.0 ± 1.4
Fluoxetine + Amlodipine	40.5 ± 0.7	26.0 ± 0.0	43.5 ± 1.8
Fluoxetine + Escitalopram	41.0 ± 1.4	28.5 ± 1.1	46.5 ± 0.7
Amlodipine + Escitalopram	34.5 ± 1.4	29.5 ± 1.1	34.0 ± 2.5

Values are mean ± standard deviation from two independent determinations (*n* = 2). Vehicle = ≤ 2% (v/v) ethanol in water without drug. Drug stocks were prepared in 96% ethanol; the final in-well ethanol concentration was ≤ 2% (v/v) and solvent-matched across arms. Values are inhibition-zone diameters measured on 150-mm plates. Diffusion results are screening outcomes and do not define synergy. Drug-source tablet strengths were amlodipine 20 mg, ciprofloxacin 250 mg, escitalopram 40 mg, fluconazole 200 mg, and fluoxetine 40 mg

#### Dual combinations against the clinical *P. aeruginosa* isolate

For the *P. aeruginosa* clinical isolate, the most pronounced inhibition zones were achieved by ciprofloxacin-containing dual combinations, particularly amlodipine + ciprofloxacin (47.5 ± 0.7 mm) and ciprofloxacin + escitalopram (46.5 ± 2.1 mm), with ciprofloxacin + fluconazole also remaining high (42.0 ± 0.0 mm). Relative to monotherapy, amlodipine + ciprofloxacin exceeded amlodipine alone (20.0 ± 0.0 mm) and increased inhibition beyond ciprofloxacin alone (39.0 ± 4.2 mm). In contrast, some fluconazole-containing dual combinations without ciprofloxacin showed limited inhibition (e.g., amlodipine + fluconazole, 15.0 ± 0.0 mm; fluconazole + escitalopram, 16.0 ± 0.0 mm), whereas non-antibiotic pairs retained moderate activity (e.g., amlodipine + escitalopram, 29.5 ± 1.1 mm; fluoxetine + escitalopram, 28.5 ± 1.1 mm; fluoxetine + amlodipine, 26.0 ± 0.0 mm). These diffusion-based trends are summarized in Table 2.

#### Dual combinations against the clinical *C. albicans* isolate

Against the *C. albicans* clinical isolate, fluoxetine + fluconazole produced the largest inhibition zone (50.5 ± 1.8 mm). Several additional fluconazole-containing combinations also increased inhibition relative to fluconazole monotherapy (30.0 ± 0.0 mm), including amlodipine + fluconazole (45.5 ± 0.7 mm) and fluconazole + escitalopram (44.0 ± 1.4 mm). Non-fluconazole combinations also showed substantial zones (e.g., fluoxetine + escitalopram, 46.5 ± 0.7 mm; fluoxetine + ciprofloxacin, 44.0 ± 1.4 mm). Diffusion results are summarized in Table 2.

### Efficacy of triplet combinations

Triplet regimens produced the largest diffusion zones in the screening assay, although the combination with the largest zone differed by organism. Because zone diameters are affected by diffusion and physicochemical properties, these findings are reported as phenotypic enhancement outcomes; formal interaction classification was based on checkerboard FICI results.

#### Triplet combinations against the clinical *S. aureus* isolate

Against the clinical *S. aureus* isolate, ciprofloxacin + fluoxetine + amlodipine produced the largest diffusion zone (101.0 ± 2.8 mm), followed by ciprofloxacin + amlodipine + fluconazole (95.5 ± 3.5 mm). These triplets produced larger zones than the corresponding single agents and dual combinations in the diffusion screen (Table 3).

**Table 3:** Inhibition-zone diameters (mm) of triplet combinations in agar-well diffusion screening against clinical isolates of *Staphylococcus aureus*, *Pseudomonas aeruginosa*, and *Candida albicans*

Combination	*Staphylococcus aureus* clinical isolate	*Pseudomonas aeruginosa* clinical isolate	*Candida albicans* clinical isolate
Vehicle (≤ 2% EtOH in water)	0.0 ± 0.0	0.0 ± 0.0	0.0 ± 0.0
Ciprofloxacin + Fluoxetine + Amlodipine	101.0 ± 2.8	80.0 ± 2.8	107.0 ± 1.4
Ciprofloxacin + Fluoxetine + Escitalopram	92.0 ± 5.7	88.5 ± 3.5	87.0 ± 2.8
Ciprofloxacin + Fluconazole + Fluoxetine	99.0 ± 2.8	58.5 ± 2.1	126.5 ± 0.7
Ciprofloxacin + Fluconazole + Amlodipine	95.5 ± 3.5	66.0 ± 1.4	138.5 ± 2.1
Ciprofloxacin + Amlodipine + Escitalopram	90.0 ± 1.4	77.5 ± 3.5	91.5 ± 2.1
Ciprofloxacin + Fluconazole + Escitalopram	74.0 ± 4.2	52.0 ± 0.0	97.5 ± 0.7
Fluconazole + Fluoxetine + Amlodipine	86.5 ± 2.1	47.0 ± 1.4	134.0 ± 0.0
Fluconazole + Amlodipine + Escitalopram	76.5 ± 0.7	38.0 ± 1.4	116.5 ± 0.7
Fluoxetine + Amlodipine + Escitalopram	82.5 ± 0.7	62.0 ± 1.4	98.5 ± 0.7

Values are mean ± standard deviation from two independent determinations (*n* = 2). Vehicle = ≤ 2% (v/v) ethanol in water without drug. Drug stocks were prepared in 96% ethanol; the final in-well ethanol concentration was ≤ 2% (v/v) and solvent-matched across arms. Diffusion results are screening outcomes and do not define synergy. Drug-source tablet strengths were amlodipine 20 mg, ciprofloxacin 250 mg, escitalopram 40 mg, fluconazole 200 mg, and fluoxetine 40 mg

#### Triplet combinations against the clinical *P. aeruginosa* isolate

For the clinical *P. aeruginosa* isolate, ciprofloxacin + fluoxetine + escitalopram produced the largest diffusion zone (88.5 ± 3.5 mm), followed by ciprofloxacin + fluoxetine + amlodipine (80.0 ± 2.8 mm). Triplets without ciprofloxacin produced smaller zones in this screening assay (Table 3).

#### Triplet combinations against the clinical *C. albicans* isolate

Against the clinical *C. albicans* isolate, ciprofloxacin + amlodipine + fluconazole produced the largest diffusion zone (138.5 ± 2.1 mm). Amlodipine + fluoxetine + fluconazole (134.0 ± 0.0 mm) and ciprofloxacin + fluoxetine + fluconazole (126.5 ± 0.7 mm) also produced large zones. These findings indicate pronounced diffusion-based enhancement in selected fluconazole-containing triplets (Table 3).

### MIC, MBC/MFC, and FIC determinations

For the *S. aureus* reference strain, ciprofloxacin was the most active single agent (MIC = 31.25 µg mL⁻¹; MBC = 500 µg mL⁻¹), whereas the non-antibiotic agents had higher MIC and MBC values (125–2000 µg mL⁻¹). When ciprofloxacin was combined with two repurposed drugs, the MIC decreased 64-fold to 0.488 µg mL⁻¹ and the MBC to 7.81 µg mL⁻¹. All four tested triplets yielded ΣFIC values of 0.14, meeting the prespecified criterion for synergy.

For *P. aeruginosa* ATCC, ciprofloxacin alone had an MIC and MBC of 1.95 µg mL⁻¹, whereas amlodipine, fluoxetine, and fluconazole required concentrations of at least 125 µg mL⁻¹. In the tested triplets, the ciprofloxacin MIC decreased fourfold to 0.488 µg mL⁻¹. The corresponding ΣFIC values were 0.320 for combinations containing escitalopram and 0.375 for combinations containing amlodipine, meeting the criterion for synergy.

Against the clinical *C. albicans* isolate, fluconazole alone had an MIC of 500 µg mL⁻¹. Triplets containing fluconazole with amlodipine plus fluoxetine or ciprofloxacin plus fluoxetine reduced the MIC to 7.81 µg mL⁻¹ and the MFC to 125 µg mL⁻¹, with ΣFIC values of 0.383 and 0.273, respectively. In contrast, the tested combinations in which fluoxetine was replaced by amlodipine or escitalopram yielded ΣFIC values of 1.13 and 2.26 and were classified as indifferent.

Taken together, the MIC and MBC/MFC reductions and the ΣFIC results indicate checkerboard-defined synergy for selected ciprofloxacin-centered combinations against *S. aureus* and *P. aeruginosa* and for selected fluconazole–fluoxetine combinations against *C. albicans*. Further details are shown in Table 4.

**Table 4:** Minimum inhibitory concentration, minimum bactericidal or fungicidal concentration, and fractional inhibitory concentration index results for single agents and triplet combinations

Organism	Regimen	Agent or combination	Component	Initial concentration (µg/mL)	MIC (µg/mL)	MBC/MFC (µg/mL)	FICI (ΣFIC)	Interpretation
*Staphylococcus aureus* ATCC 29,213	Single	Ciprofloxacin	-	1000	31.25	500	Not applicable	Not applicable
*Staphylococcus aureus* ATCC 29,213	Single	Amlodipine	-	4000	125	2000	Not applicable	Not applicable
*Staphylococcus aureus* ATCC 29,213	Single	Fluoxetine	-	4000	125	2000	Not applicable	Not applicable
*Staphylococcus aureus* ATCC 29,213	Single	Fluconazole	-	4000	125	Not determined	Not applicable	Not applicable
*Staphylococcus aureus* ATCC 29,213	Single	Escitalopram	-	4000	125	Not determined	Not applicable	Not applicable
*Staphylococcus aureus* ATCC 29,213	Combination	Ciprofloxacin, Escitalopram, Fluoxetine	Cip	250	0.488	0.488	0.141	Synergy
*Staphylococcus aureus* ATCC 29,213			Esc	4000	7.81	7.81		
*Staphylococcus aureus* ATCC 29,213			FX	4000	7.81	7.81		
*Staphylococcus aureus* ATCC 29,213	Combination	Ciprofloxacin, Escitalopram, Amlodipine	Cip	250	0.488	0.488	0.141	Synergy
*Staphylococcus aureus* ATCC 29,213			Esc	4000	7.81	7.81		
*Staphylococcus aureus* ATCC 29,213			Am	4000	7.81	7.81		
*Staphylococcus aureus* ATCC 29,213	Combination	Ciprofloxacin, Fluoxetine, Amlodipine	Cip	250	0.488	0.488	0.141	Synergy
*Staphylococcus aureus* ATCC 29,213			FX	4000	7.81	7.81		
*Staphylococcus aureus* ATCC 29,213			Am	4000	7.81	7.81		
*Staphylococcus aureus* ATCC 29,213	Combination	Ciprofloxacin, Fluoxetine, Fluconazole	Cip	250	0.488	0.488	0.141	Synergy
*Staphylococcus aureus* ATCC 29,213			FX	4000	7.81	7.81		
*Staphylococcus aureus* ATCC 29,213			FZ	4000	7.81	7.81		
*Pseudomonas aeruginosa* ATCC 27,853	Single	Ciprofloxacin	-	1000	1.95	1.95	Not applicable	Not applicable
*Pseudomonas aeruginosa* ATCC 27,853	Single	Amlodipine	-	4000	125	1000	Not applicable	Not applicable
*Pseudomonas aeruginosa* ATCC 27,853	Single	Fluoxetine	-	4000	125	125	Not applicable	Not applicable
*Pseudomonas aeruginosa* ATCC 27,853	Single	Fluconazole	-	4000	125	2000	Not applicable	Not applicable
*Pseudomonas aeruginosa* ATCC 27,853	Single	Escitalopram	-	4000	1000	2000	Not applicable	Not applicable
*Pseudomonas aeruginosa* ATCC 27,853	Combination	Ciprofloxacin, Escitalopram, Fluoxetine	Cip	250	0.488	0.488	0.321	Synergy
*Pseudomonas aeruginosa* ATCC 27,853			Esc	4000	7.81	7.81		
*Pseudomonas aeruginosa* ATCC 27,853			FX	4000	7.81	7.81		
*Pseudomonas aeruginosa* ATCC 27,853	Combination	Ciprofloxacin, Escitalopram, Amlodipine	Cip	250	0.488	0.488	0.321	Synergy
*Pseudomonas aeruginosa* ATCC 27,853			Esc	4000	7.81	7.81		
*Pseudomonas aeruginosa* ATCC 27,853			Am	4000	7.81	7.81		
*Pseudomonas aeruginosa* ATCC 27,853	Combination	Ciprofloxacin, Fluoxetine, Amlodipine	Cip	250	0.488	0.488	0.375	Synergy
*Pseudomonas aeruginosa* ATCC 27,853			FX	4000	7.81	7.81		
*Pseudomonas aeruginosa* ATCC 27,853			Am	4000	7.81	7.81		
*Pseudomonas aeruginosa* ATCC 27,853	Combination	Ciprofloxacin, Fluoxetine, Fluconazole	Cip	250	0.488	0.488	0.375	Synergy
*Pseudomonas aeruginosa* ATCC 27,853			FX	4000	7.81	7.81		
*Pseudomonas aeruginosa* ATCC 27,853			FZ	4000	7.81	7.81		
*Candida albicans* clinical isolate	Single	Ciprofloxacin	-	1000	31.25	500	Not applicable	Not applicable
*Candida albicans* clinical isolate	Single	Amlodipine	-	4000	62.5	1000	Not applicable	Not applicable
*Candida albicans* clinical isolate	Single	Fluoxetine	-	4000	31.25	250	Not applicable	Not applicable
*Candida albicans* clinical isolate	Single	Fluconazole	-	4000	500	2000	Not applicable	Not applicable
*Candida albicans* clinical isolate	Single	Escitalopram	-	4000	1000	2000	Not applicable	Not applicable
*Candida albicans* clinical isolate	Combination	Fluconazole, Amlodipine, Fluoxetine	FZ	4000	7.81	125	0.391	Synergy
*Candida albicans* clinical isolate			Am	4000	7.81	125		
*Candida albicans* clinical isolate			FX	4000	7.81	125		
*Candida albicans* clinical isolate	Combination	Fluconazole, Ciprofloxacin, Fluoxetine	FZ	4000	7.81	125	0.281	Synergy
*Candida albicans* clinical isolate			Cip	250	0.488	7.8		
*Candida albicans* clinical isolate			FX	4000	7.81	125		
*Candida albicans* clinical isolate	Combination	Ciprofloxacin, Amlodipine, Fluoxetine	Cip	250	3.9	7.81	3.125	Indifferent
*Candida albicans* clinical isolate			Am	4000	62.5	125		
*Candida albicans* clinical isolate			FX	4000	62.5	125		
*Candida albicans* clinical isolate	Combination	Ciprofloxacin, Amlodipine, Escitalopram	Cip	250	7.81	Not determined	2.375	Indifferent
*Candida albicans* clinical isolate			Am	4000	125	250		
*Candida albicans* clinical isolate			Esc	4000	125	250		

*MIC *minimum inhibitory concentration,* MBC* minimum bactericidal concentration, *MFC* minimum fungicidal concentration, *FIC* fractional inhibitory concentration, *FICI*  fractional inhibitory concentration index, ΣFIC = FIC of drug A + FIC of drug B + FIC of drug C for triplet combinations. *Cip* ciprofloxacin, *Esc *escitalopram, *FX* fluoxetine, *FZ* fluconazole, *Am*  amlodipine. FICI interpretation: synergy, ≤ 0.5; additivity, > 0.5 to 1; indifference, > 1 to 4; antagonism, > 4. “Not determined” indicates that a reliable endpoint was not available for reporting. Drug-source tablet strengths were amlodipine 20 mg, ciprofloxacin 250 mg, escitalopram 40 mg, fluconazole 200 mg, and fluoxetine 40 mg

### Molecular docking results

**Fig. 1:**
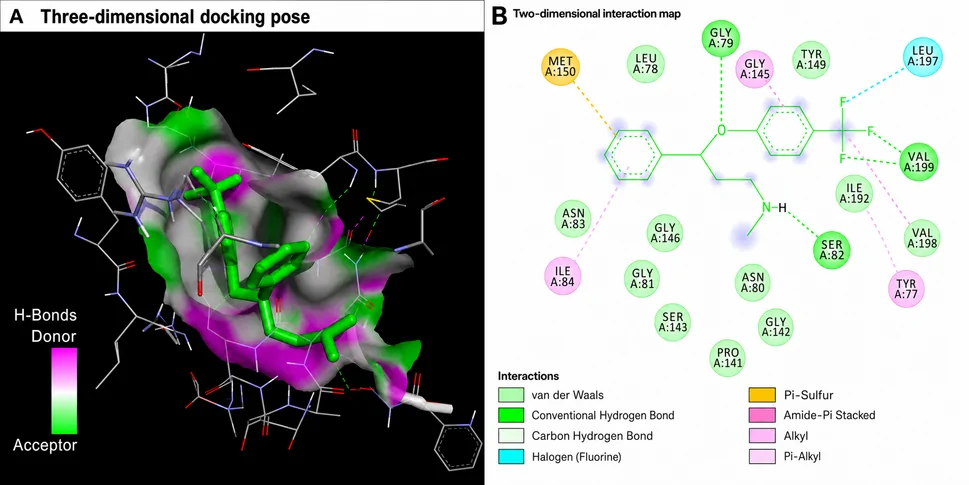
Molecular docking of fluoxetine in the active site of *Staphylococcus aureus* MurB (PDB: 1HSK). **A** Three-dimensional binding pose of fluoxetine within the NADPH-binding pocket of MurB. **B** Two-dimensional interaction map showing the main contacts formed between fluoxetine and surrounding amino-acid residues. The calculated binding free energy was − 8.6 kcal mol⁻¹

**Fig. 2:**
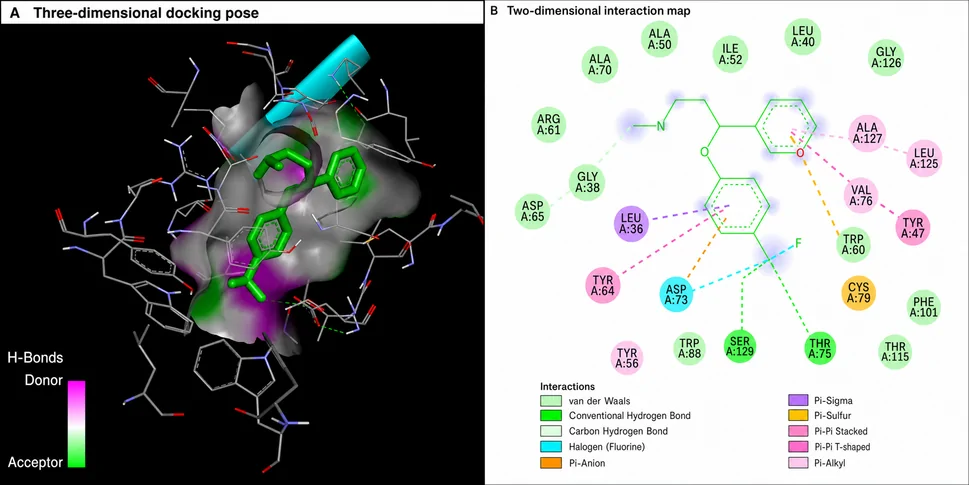
Molecular docking of fluoxetine in the ligand-binding pocket of *Pseudomonas aeruginosa* LasR (PDB: 3IX3). **A** Three-dimensional binding pose of fluoxetine within the LasR binding cavity. **B** Two-dimensional interaction map illustrating the principal hydrogen-bonding, hydrophobic, and π-related interactions with the surrounding residues. The calculated binding free energy was − 9.1 kcal mol⁻¹

**Fig. 3:**
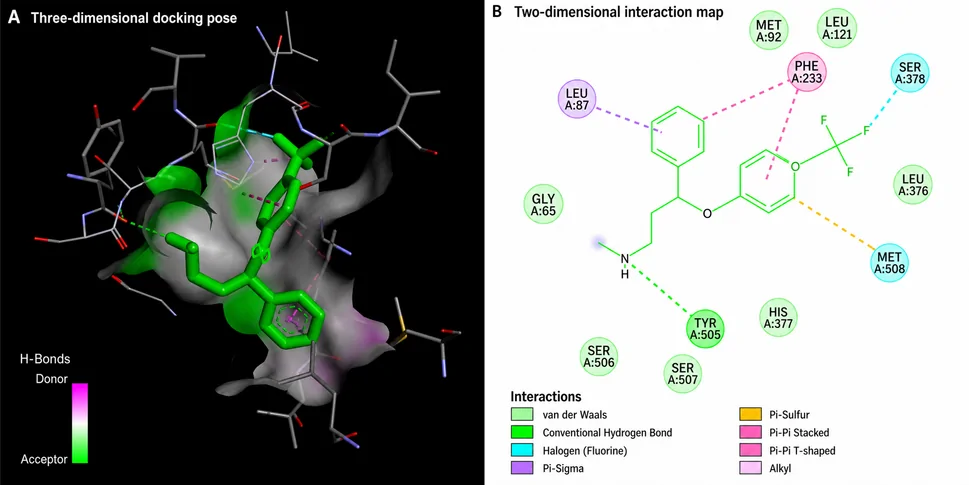
Molecular docking of fluoxetine in the active site of *Candida albicans* lanosterol 14-α-demethylase (CYP51; PDB: 5V5Z). **A** Three-dimensional binding pose of fluoxetine positioned above the heme-containing active site. **B** Two-dimensional interaction map showing the key contacts between fluoxetine and the surrounding amino-acid residues. The calculated binding free energy was − 8.2 kcal mol⁻¹

Molecular docking was performed against three pathogen-specific targets: MurB from *S. aureus* (PDB 1HSK), LasR from *P. aeruginosa* (PDB 3IX3), and lanosterol 14α-demethylase (CYP51) from *C. albicans* (PDB 5V5Z). The complete set of docking poses and interaction maps is provided in Supplementary Fig. S1–S15; the selected fluoxetine complexes are shown in Figs. 1, 2 and 3.

For MurB, fluoxetine yielded a predicted docking score of − 8.6 kcal mol⁻¹ (Fig. 1). Ciprofloxacin yielded a slightly more favorable score but showed an unfavorable donor–donor contact in the modeled pose. These calculations support plausible occupancy of the modeled pocket but do not establish binding affinity, target occupancy, or mechanism.

For LasR, fluoxetine yielded the most favorable predicted docking score among the tested compounds (− 9.1 kcal mol⁻¹; Fig. 2). The modeled pose included hydrogen-bonding contacts with SER235 and TYR42 and π-related interactions with tyrosine residues. Ciprofloxacin and fluconazole yielded predicted scores of − 8.8 and − 8.5 kcal mol⁻¹, respectively. These docking outputs were interpreted as structural hypotheses rather than measurements of binding affinity.

For CYP51, fluoxetine yielded a predicted docking score of − 8.2 kcal mol⁻¹ (Fig. 3), close to the fluconazole score of − 8.3 kcal mol⁻¹. The modeled fluoxetine pose included a hydrogen-bonding contact with TYR505 and halogen-related contacts with SER378 and MET508. Amlodipine yielded a predicted score of − 6.8 kcal mol⁻¹ and formed four modeled hydrogen bonds. These poses provide hypotheses for potential target-associated interactions but do not demonstrate target engagement.

Across the three modeled targets, fluoxetine yielded favorable predicted docking scores and multiple modeled contacts. The docking results were compatible with a hypothesis that fluoxetine may interact with more than one pathogen-associated target; experimental binding, target engagement, and causal mechanisms remain unconfirmed.

### Normal mode analysis (NMA) of selected docked complexes

To compare predicted collective motions in the selected docked models, NMA was performed for fluoxetine bound to *P. aeruginosa* LasR (PDB 3IX3) and *S. aureus* MurB (PDB 1HSK). The outputs included molecular mobility, deformability, estimated B-factors, eigenvalues, variance, covariance maps, and elastic network models.

**Fig. 4:**
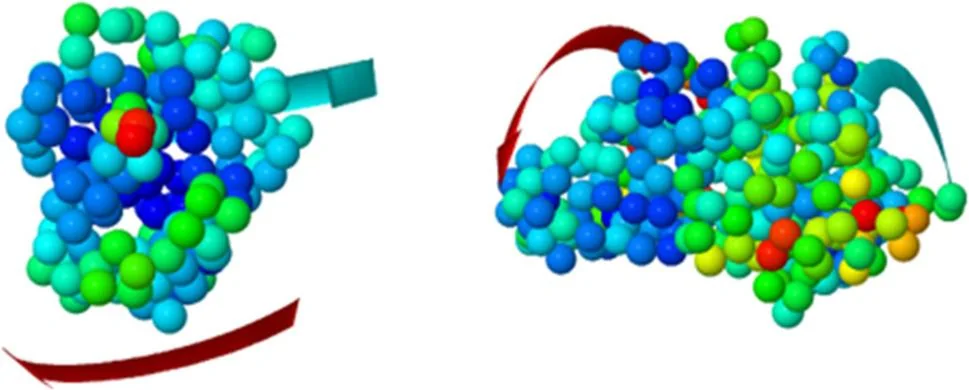
Molecular mobility analysis of the selected docked complexes obtained by normal mode analysis (NMA): **A** 3IX3–fluoxetine complex and **B** 1HSK–fluoxetine complex. The arrows indicate the direction and relative magnitude of motion within each complex

The mobility analysis indicated coordinated predicted motion in both complexes. The 3IX3–fluoxetine model showed broader collective displacement and greater directional movement, consistent with comparatively greater predicted conformational flexibility. The 1HSK–fluoxetine model showed more restricted and localized motion (Fig. 4).

**Fig. 5:**
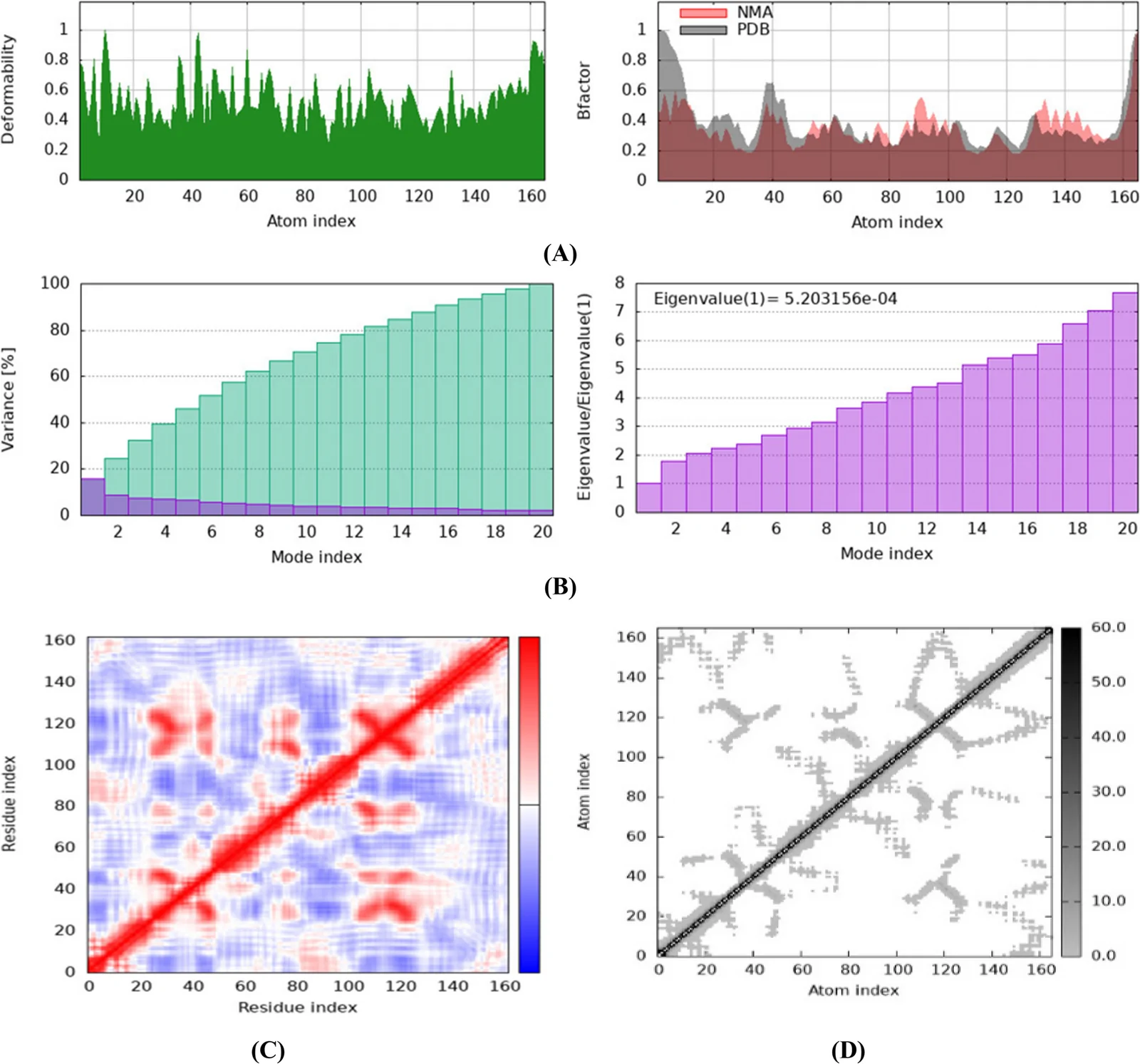
Normal mode analysis outputs for the 3IX3–fluoxetine complex: **A** deformability and B-factor plot, **B** eigenvalue and variance plot, **C** elastic network model, and **D** covariance map

**Fig. 6:**
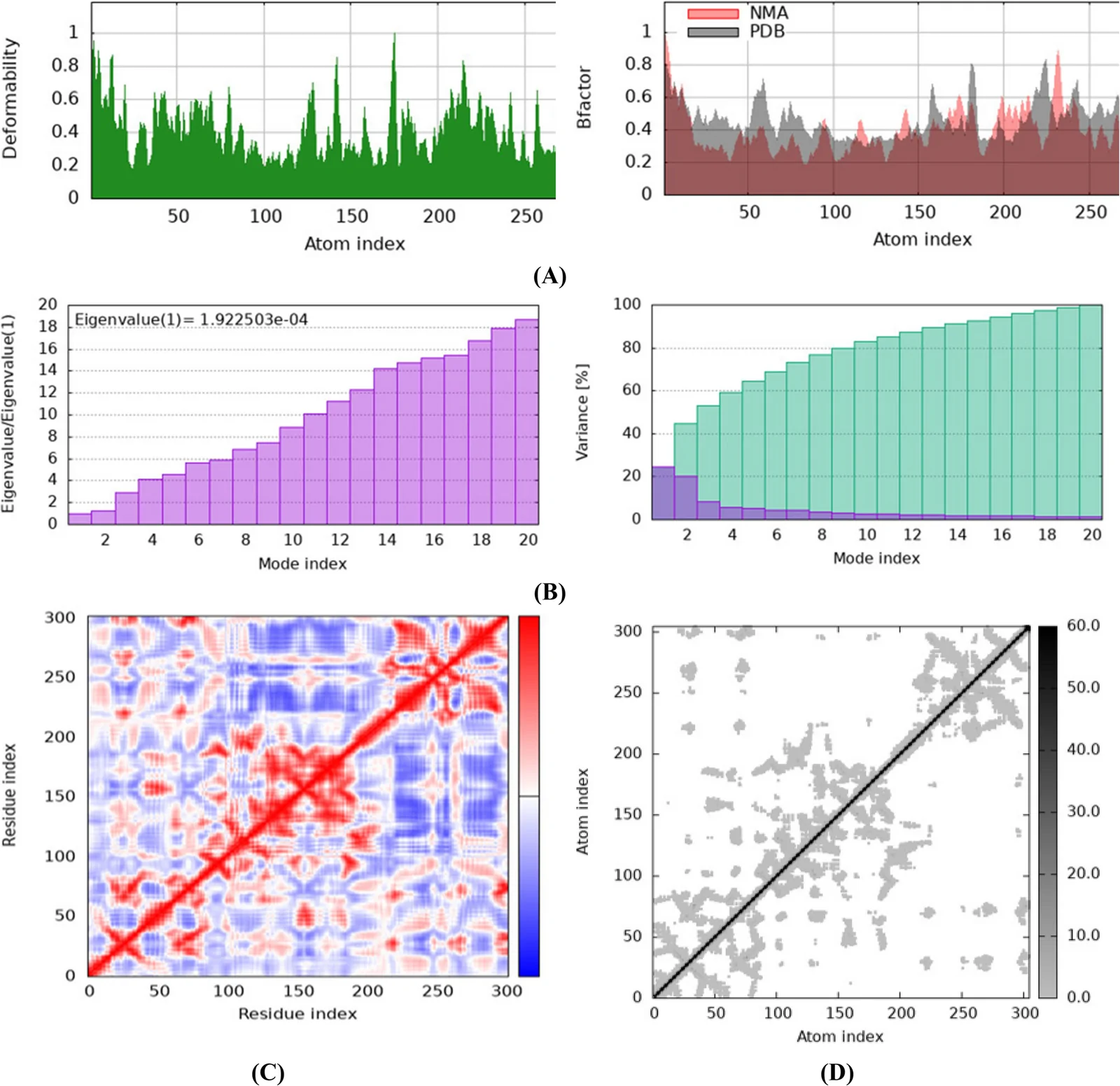
Normal mode analysis outputs for the 1HSK–fluoxetine complex: **A** deformability and B-factor plot, **B** eigenvalue and variance plot, **C** elastic network model, and **D** covariance map

Deformability analysis showed relatively low predicted deformability across most regions of both models, with localized flexible segments. The 3IX3–fluoxetine model showed more pronounced local deformability peaks, whereas the 1HSK–fluoxetine model showed lower predicted deformability overall (Figs. 5A and 6A).

The estimated B-factor profiles were consistent with the deformability outputs. The 3IX3–fluoxetine model showed a broader distribution of predicted mobility, whereas the 1HSK–fluoxetine model showed a narrower fluctuation profile (Figs. 5A and 6A).

The eigenvalue results further differentiate between the two models. For the 3IX3–fluoxetine model, the first ten modes ranged from 5.20 × 10⁻⁴ to 2.00 × 10⁻³, indicating lower modeled resistance to collective motion. For the 1HSK–fluoxetine model, eigenvalues ranged from 8.72 × 10⁻⁴ to 1.08 × 10⁻³, indicating a more constrained modeled motion pattern (Figs. 5B and 6B).

Variance analysis indicated greater cumulative and individual-mode variance for the 3IX3–fluoxetine model and lower variance for the 1HSK–fluoxetine model (Figs. 5B and 6B).

Covariance mapping showed broader regions of correlated and anti-correlated predicted motion in the 3IX3–fluoxetine model and a more compact covariance pattern in the 1HSK–fluoxetine model (Figs. 5D and 6D).

The elastic network output showed a denser and more uniformly distributed network of modeled inter-residue connections for the 1HSK–fluoxetine model than for the 3IX3–fluoxetine model (Figs. 5C and 6C).

Overall, the NMA outputs indicated distinct predicted collective-motion patterns for the two modeled complexes. The 3IX3–fluoxetine model showed greater predicted flexibility and broader collective movement, whereas the 1HSK–fluoxetine model showed more restricted and coordinated motion. These findings do not establish thermodynamic stability or experimental binding.

## Discussion

Escalating antimicrobial resistance motivates investigation of licensed non-antibiotic agents as potential adjuvants to established antimicrobial therapy. In line with prior reports of intrinsic antibacterial or antifungal activity for fluoxetine, amlodipine, and escitalopram [13, 32, 33], each agent produced measurable inhibition of *S. aureus*, *P. aeruginosa*, and *C. albicans* at the tested concentrations. Broader evidence that non-antibiotic drugs may interact with conventional antimicrobials supports the adjuvant concept [34]. Experimental studies have also suggested activity of calcium-channel-blocker scaffolds against *P. aeruginosa* [35], while antidepressants and related compounds have been investigated as efflux-pump modulators or disruptors of membrane energetics [36–38].

Dual non-antibiotic pairings, particularly fluoxetine + amlodipine and fluoxetine + escitalopram, produced enhanced inhibition zones in the diffusion screen. These findings are consistent with reports that fluoxetine can augment antibacterial activity, including activity with fluoroquinolones [13, 39], and with reports of antimicrobial or adjuvant activity for amlodipine [16, 32, 35]. When ciprofloxacin or fluconazole was incorporated into selected combinations, MIC values decreased by 4- to 64-fold and checkerboard ΣFIC values met the prespecified criterion for synergy. These findings extend earlier reports of fluoxetine–ciprofloxacin potentiation and broader non-antibiotic–antimicrobial interactions [7, 13, 34, 39], and are compatible with studies of azole combinations with non-antibiotic agents [15, 40].

For bacterial isolates, ciprofloxacin acts principally through DNA gyrase and topoisomerase IV [41]. Fluoxetine and escitalopram may increase intracellular antimicrobial exposure through effects on efflux or membrane function [36, 37, 39]. Amlodipine has demonstrated antimicrobial activity in experimental studies [16, 32, 35], but the mechanism underlying its contribution to the observed combinations was not tested. Direct membrane-potential assays would be required to evaluate membrane-related hypotheses [42]. For *C. albicans*, fluconazole inhibits Erg11/CYP51 and is usually fungistatic. Previous studies have reported synergy between fluconazole and fluoxetine or calcium-channel blockers through effects on tolerance, efflux, virulence, or calcium signaling [15, 40]. The docking results in the present study provide structural hypotheses that are compatible with these observations but do not confirm the proposed mechanisms.

MIC versus MBC/MFC. A reduction in MIC without a corresponding change in MBC or MFC may indicate enhanced growth inhibition without increased killing, whereas concurrent decreases in MIC and MBC/MFC are compatible with enhanced bactericidal or fungicidal activity [43, 44]. In the present dataset, selected combinations reduced both inhibitory and bactericidal or fungicidal endpoints. These patterns should be interpreted cautiously because time-kill experiments were not performed.

Non-antibiotic adjuvants may resensitize difficult-to-treat pathogens by affecting permeability, efflux, membrane signaling, or stress responses [15, 37, 40]. Clinical translation remains uncertain because the antimicrobial concentrations tested may not be achievable safely in patients, and cardiovascular, neuropsychiatric, and pharmacokinetic interactions would require careful evaluation. The checkerboard-defined synergy observed for selected combinations warrants confirmation in pharmacodynamic, toxicity, and in vivo studies.

The NMA outputs added a preliminary structural-dynamics perspective to the docking analysis. The 3IX3–fluoxetine model showed greater predicted conformational adaptability, whereas the 1HSK–fluoxetine model showed more restricted and coordinated motion. These target-dependent patterns are compatible with differences between a quorum-sensing regulator and a catalytic cell-wall biosynthesis enzyme. They remain computational observations and cannot establish binding affinity, biological target engagement, or causal mechanism [31, 45, 46].

Several limitations should be considered. The study evaluated a limited set of local clinical isolates and ATCC reference strains from a single laboratory, which restricts generalizability across strains, resistance phenotypes, and settings. Agar-well diffusion was used only as a screening method, and checkerboard interaction classifications were not confirmed by time-kill assays. The tested concentrations of the repurposed drugs may exceed clinically achievable unbound exposures, and cytotoxicity, pharmacokinetics, drug–drug interactions, and in vivo efficacy were not assessed. Docking and NMA were hypothesis-generating analyses; NMA is not equivalent to all-atom molecular dynamics, and no experimental binding or target-engagement studies were performed. These limitations preclude clinical inference and support the need for confirmatory mechanistic, safety, pharmacodynamic, and in vivo studies.

## Conclusion

This study shows that fluoxetine, amlodipine, and escitalopram have in vitro antimicrobial activity against *S. aureus*, *P. aeruginosa*, and *C. albicans* at the tested concentrations. Selected combinations with ciprofloxacin or fluconazole met the prespecified checkerboard criterion for synergy and reduced MIC values in an organism- and combination-dependent manner. Molecular docking and NMA provided supportive, hypothesis-generating structural context but did not establish target engagement or mechanism. Pharmacokinetic, safety, mechanistic, and in vivo validation is required before clinical application can be considered.

## Supplementary Information

Supplementary Material 1. S1–S3: 3D binding poses and 2D interaction maps of ciprofloxacin, escitalopram and amlodipine in the active site of S. aureus MurB (PDB 1HSK). S4–S6: Docking results for ciprofloxacin, escitalopram and amlodipine with P. aeruginosa LasR (PDB 3IX3). S7–S9: Ciprofloxacin, escitalopram, and amlodipine docking poses in C. albicans CYP51 (PDB 5V5Z).S10–S15: Additional two- and three-dimensional interaction maps for the remaining drug–target complexes.

Supplementary Material 2. Table S1: Reagents and media formulations used throughout the study. Table S2: Instrumentation specifications (manufacturers and model details). Table S3–S4: Serial-dilution schemes for non-antibiotic drugs (amlodipine, fluoxetine, escitalopram, fluconazole) and for ciprofloxacin. Table S5–S6: Preparation and dilution protocols for all dual and triple drug combinations. Table S7–S11: Zone-of-inhibition measurements for individual, dual and triplet regimens against clinical and ATCC strains of S. aureus, P. aeruginosa and C. albicans. Table S12–S14: Calculated binding energies, hydrogen-bond counts and key interacting residues from molecular docking to MurB (1HSK), LasR (3IX3) and CYP51 (5V5Z).

## Data Availability

The raw datasets underlying all figures and tables (agar well-diffusion zone diameters, minimum inhibitory concentration (MIC)/minimum bactericidal concentration (MBC)/minimum fungicidal concentration (MFC) values, and fractional inhibitory concentration (ΣFIC) matrices), along with plate-map templates and GraphPad Prism files, are available from the co-corresponding author on reasonable request: Wafa M. Al-Madhagi. Tel.: +967-733744714 **.** Email: w.almadhagi@su.edu.ye .

## Abbreviations

AMR: Antimicrobial resistance
ATCC: American type culture collection
CAMHB: Cation-adjusted Mueller–Hinton broth
CFU: Colony-forming unit
CLED: Cystine–lactose–electrolyte-deficient
CYP51: Lanosterol 14α-demethylase
DMSO: Dimethyl sulfoxide
FIC: Fractional inhibitory concentration
FICI: Fractional inhibitory concentration index
MBC: Minimum bactericidal concentration
MDR: Multidrug-resistant
MD: Molecular dynamics
MFC: Minimum fungicidal concentration
MIC: Minimum inhibitory concentration
MurB: UDP-N-acetylenolpyruvylglucosamine reductase
NADPH: Nicotinamide adenine dinucleotide phosphate, reduced form
NCCLS: National committee for clinical laboratory standards
NCPHL: National Center of Public Health Laboratories
NMA: Normal mode analysis
OD: Optical density
PDB: Protein data bank
QC: Quality control
SSRI: Selective serotonin reuptake inhibitor
