# Supplementary material for: Repurposing amlodipine, fluoxetine, and escitalopram as non-antibiotic adjuvants: in vitro enhancement of ciprofloxacin and fluconazole activity against multidrug-resistant bacterial and candida clinical isolates
Source: BMC Microbiol. 2026 Jul 23;26:901. doi: 10.1186/s12866-026-05443-4 (PMC13679258; doi:10.1186/s12866-026-05443-4)
Supplement: Supplementary file 1 [file 12866_2026_5443_MOESM1_ESM.docx]

**a**

**b**

**Figure S1.** Molecular docking of fluoxetine in the binding site of *Staphylococcus aureus* MurB (PDB ID: 1HSK). **(a)** Three-dimensional binding pose showing the docked orientation within the protein binding pocket. **(b)** Two-dimensional interaction map showing the principal hydrogen-bonding and other non-covalent contacts with surrounding amino-acid residues. The calculated binding energy was −8.6 kcal/mol.

**a**

**b**

**Figure S2.** Molecular docking of ciprofloxacin in the binding site of *Staphylococcus aureus* MurB (PDB ID: 1HSK). **(a)** Three-dimensional binding pose showing the docked orientation within the protein binding pocket. **(b)** Two-dimensional interaction map showing the principal hydrogen-bonding and other non-covalent contacts with surrounding amino-acid residues. The calculated binding energy was −9.1 kcal/mol.

**b**

**a**

**Figure S3.** Molecular docking of amlodipine in the binding site of *Staphylococcus aureus* MurB (PDB ID: 1HSK). **(a)** Three-dimensional binding pose showing the docked orientation within the protein binding pocket. **(b)** Two-dimensional interaction map showing the principal hydrogen-bonding and other non-covalent contacts with surrounding amino-acid residues. The calculated binding energy was −6.8 kcal/mol.

**a**

**b**

**Figure S4.** Molecular docking of escitalopram in the binding site of *Staphylococcus aureus* MurB (PDB ID: 1HSK). **(a)** Three-dimensional binding pose showing the docked orientation within the protein binding pocket. **(b)** Two-dimensional interaction map showing the principal hydrogen-bonding and other non-covalent contacts with surrounding amino-acid residues. The calculated binding energy was −7.7 kcal/mol.

**a**

**b**

**Figure S5.** Molecular docking of fluconazole in the binding site of *Staphylococcus aureus* MurB (PDB ID: 1HSK). **(a)** Three-dimensional binding pose showing the docked orientation within the protein binding pocket. **(b)** Two-dimensional interaction map showing the principal hydrogen-bonding and other non-covalent contacts with surrounding amino-acid residues. The calculated binding energy was −8.4 kcal/mol.

**a**

**Figure S6.** Molecular docking of ciprofloxacin in the binding site of *Pseudomonas aeruginosa* LasR (PDB ID: 3IX3). **(a)** Three-dimensional binding pose showing the docked orientation within the protein binding pocket. **(b)** Two-dimensional interaction map showing the principal hydrogen-bonding and other non-covalent contacts with surrounding amino-acid residues. The calculated binding energy was −8.8 kcal/mol.

**b**

**a**

**Figure S7.** Molecular docking of fluconazole in the binding site of *Pseudomonas aeruginosa* LasR (PDB ID: 3IX3). **(a)** Three-dimensional binding pose showing the docked orientation within the protein binding pocket. **(b)** Two-dimensional interaction map showing the principal hydrogen-bonding and other non-covalent contacts with surrounding amino-acid residues. The calculated binding energy was −8.5 kcal/mol.

**b**

**a**

**Figure S8.** Molecular docking of escitalopram in the binding site of *Pseudomonas aeruginosa* LasR (PDB ID: 3IX3). **(a)** Three-dimensional binding pose showing the docked orientation within the protein binding pocket. **(b)** Two-dimensional interaction map showing the principal hydrogen-bonding and other non-covalent contacts with surrounding amino-acid residues. The calculated binding energy was −6.8 kcal/mol.

**b**

**a**

**Figure S9.** Molecular docking of amlodipine in the binding site of *Pseudomonas aeruginosa* LasR (PDB ID: 3IX3). **(a)** Three-dimensional binding pose showing the docked orientation within the protein binding pocket. **(b)** Two-dimensional interaction map showing the principal hydrogen-bonding and other non-covalent contacts with surrounding amino-acid residues. The calculated binding energy was −5.4 kcal/mol.

**b**

**a**

**Figure S10.** Molecular docking of fluoxetine in the binding site of *Pseudomonas aeruginosa* LasR (PDB ID: 3IX3). **(a)** Three-dimensional binding pose showing the docked orientation within the protein binding pocket. **(b)** Two-dimensional interaction map showing the principal hydrogen-bonding and other non-covalent contacts with surrounding amino-acid residues. The calculated binding energy was −9.1 kcal/mol.

**a**

**b**

**Figure S11.** Molecular docking of fluoxetine in the binding site of *Candida albicans* lanosterol 14α-demethylase (CYP51) (PDB ID: 5V5Z). **(a)** Three-dimensional binding pose showing the docked orientation within the protein binding pocket. **(b)** Two-dimensional interaction map showing the principal hydrogen-bonding and other non-covalent contacts with surrounding amino-acid residues. The calculated binding energy was −8.2 kcal/mol.

**Figure S12.** Molecular docking of fluconazole in the binding site of *Candida albicans* lanosterol 14α-demethylase (CYP51) (PDB ID: 5V5Z). **(a)** Three-dimensional binding pose showing the docked orientation within the protein binding pocket. **(b)** Two-dimensional interaction map showing the principal hydrogen-bonding and other non-covalent contacts with surrounding amino-acid residues. The calculated binding energy was −8.3 kcal/mol.

**a**

**b**

**Figure S13.** Molecular docking of amlodipine in the binding site of *Candida albicans* lanosterol 14α-demethylase (CYP51) (PDB ID: 5V5Z). **(a)** Three-dimensional binding pose showing the docked orientation within the protein binding pocket. **(b)** Two-dimensional interaction map showing the principal hydrogen-bonding and other non-covalent contacts with surrounding amino-acid residues. The calculated binding energy was −6.8 kcal/mol.

**b**

**a**

**Figure S14.** Molecular docking of ciprofloxacin in the binding site of *Candida albicans* lanosterol 14α-demethylase (CYP51) (PDB ID: 5V5Z). **(a)** Three-dimensional binding pose showing the docked orientation within the protein binding pocket. **(b)** Two-dimensional interaction map showing the principal hydrogen-bonding and other non-covalent contacts with surrounding amino-acid residues. The calculated binding energy was −8.3 kcal/mol.

**a**

**b**

**Figure S15.** Molecular docking of escitalopram in the binding site of *Candida albicans* lanosterol 14α-demethylase (CYP51) (PDB ID: 5V5Z). **(a)** Three-dimensional binding pose showing the docked orientation within the protein binding pocket. **(b)** Two-dimensional interaction map showing the principal hydrogen-bonding and other non-covalent contacts with surrounding amino-acid residues. The calculated binding energy was −8.2 kcal/mol.
