# Supplementary material for: Repurposing amlodipine, fluoxetine, and escitalopram as non-antibiotic adjuvants: in vitro enhancement of ciprofloxacin and fluconazole activity against multidrug-resistant bacterial and candida clinical isolates
Source: BMC Microbiol. 2026 Jul 23;26:901. doi: 10.1186/s12866-026-05443-4 (PMC13679258; doi:10.1186/s12866-026-05443-4)
Supplement: Supplementary file 2 [file 12866_2026_5443_MOESM2_ESM.docx]

### Laboratory requirements

**Table S1. Chemicals and culture media used in the laboratory.**

| **Material** | **Manufacturer** | **Country** |
| --- | --- | --- |
| Blood agar | OXOID | England |
| Brain-heart infusion broth | OXOID | England |
| Cystine-lactose-electrolyte-deficient (CLED) agar | OXOID | England |
| Dimethyl sulfoxide | Helitprofen | United Kingdom |
| Distilled water generated by an ELGA purification system | ELGA | UK |
| Ethanol (96%) | Sheba Pharma | Yemen |
| Ethanol (96%) | ECOCHEM | New Zealand |
| Mueller-Hinton agar | OXOID | England |
| Normal saline | Pharmaceutical Solutions Industry | Saudi Arabia |
| Nutrient broth | BIOTEC | UK |

Manufacturer and country details are reported as recorded in the laboratory inventory. CLED = cystine-lactose-electrolyte-deficient.

**Table S2. Laboratory instruments and equipment.**

| **Instrument or equipment** | **Manufacturer** | **Country** |
| --- | --- | --- |
| Autoclave | Tomy | Tokyo, Japan |
| Deep freezer | Angelantoni | Italy |
| Disposable cotton swabs | Heinz Herenz | Ireland |
| Disposable inoculating loops | Welkang | China |
| ELISA reader (Multiskan EX) | Thermo Electron Corporation | USA |
| Incubator | Memmert | Germany |
| Multichannel micropipette | Socorex | Switzerland |
| Single-channel micropipette | Thermo Labsystems | USA |
| Petri dishes | Polylab | India |
| Vertical cooler | Soistar | Turkey |

ELISA = enzyme-linked immunosorbent assay; USA = United States of America.

**Table S3. Serial dilution scheme for amlodipine, fluoxetine, escitalopram, and fluconazole.**

| **Drug concentration (µg/mL)** | **Dilution number** |
| --- | --- |
| 4000 | 1 (initial concentration) |
| 2000 | 2 |
| 1000 | 3 |
| 500 | 4 |
| 250 | 5 |
| 125 | 6 |
| 62.5 | 7 |
| 31.25 | 8 |
| 15.63 | 9 |
| 7.8 | 10 |
| Positive control (broth and microbial suspension) | 11 |
| Negative control (broth only) | 12 |

**Table S4. Serial dilution scheme for ciprofloxacin.**

| **Drug concentration (µg/mL)** | **Dilution number** |
| --- | --- |
| 1000 | 1 (initial concentration) |
| 500 | 2 |
| 250 | 3 |
| 125 | 4 |
| 62.5 | 5 |
| 31.25 | 6 |
| 15.63 | 7 |
| 7.8 | 8 |
| 3.9 | 9 |
| 1.9 | 10 |
| Positive control (broth and microbial suspension) | 11 |
| Negative control (broth only) | 12 |

Positive control = broth plus microbial suspension; negative control = broth only. Concentrations are nominal concentrations before inoculation.

**Table S5. Preparation of three-drug combination stock solutions.**

| Total volume | **Ciprofloxacin** | **Fluoxetine** | **Fluconazole** | **Components** |
| --- | --- | --- | --- | --- |
| 3 mL | 0.25 mg/mL | 4 mg/mL | 4 mg/mL | Nominal concentration |
| Total volume | **Ciprofloxacin** | **Fluoxetine** | **Amlodipine** | **Components** |
| 3 mL | 0.25 mg/mL | 4 mg/mL | 4 mg/mL | Nominal concentration |
| Total volume | **Ciprofloxacin** | **Fluoxetine** | **Escitalopram** | **Components** |
| 3 mL | 0.25 mg/mL | 4 mg/mL | 4 mg/mL | Nominal concentration |
| Total volume | **Ciprofloxacin** | **Amlodipine** | **Fluconazole** | **Components** |
| 3 mL | 0.25 mg/mL | 4 mg/mL | 4 mg/mL | Nominal concentration |
| Total volume | **Ciprofloxacin** | **Escitalopram** | **Fluconazole** | **Components** |
| 3 mL | 0.25 mg/mL | 4 mg/mL | 4 mg/mL | Nominal concentration |
| Total volume | **Ciprofloxacin** | **Escitalopram** | **Amlodipine** | **Components** |
| 3 mL | 0.25 mg/mL | 4 mg/mL | 4 mg/mL | Nominal concentration |
| Total volume | **Amlodipine** | **Fluoxetine** | **Fluconazole** | **Components** |
| 3 mL | 4 mg/mL | 4 mg/mL | 4 mg/mL | Nominal concentration |
| Total volume | **Amlodipine** | **Escitalopram** | **Fluconazole** | **Components** |
| 3 mL | 4 mg/mL | 4 mg/mL | 4 mg/mL | Nominal concentration |
| Total volume | **Amlodipine** | **Escitalopram** | **Fluoxetine** | **Components** |
| 3 mL | 4 mg/mL | 4 mg/mL | 4 mg/mL | Nominal concentration |
| Total volume | **Escitalopram** | **Fluoxetine** | **Fluconazole** | **Components** |
| 3 mL | 4 mg/mL | 4 mg/mL | 4 mg/mL | Nominal concentration |

Each row pair reports the nominal concentrations of the component stock solutions used to prepare a 3-mL triplet mixture. Ciprofloxacin was prepared at 0.25 mg/mL; the other component solutions were prepared at 4 mg/mL.

**Table S6. Serial dilution scheme for drug combinations.**

| **Concentration of each co-agent (µg/mL)** | | **Dilution number** |
| --- | --- | --- |
| **Ciprofloxacin** | **Amlodipine, fluoxetine, escitalopram, or fluconazole** |  |
| 250 | 4000 | 1 |
| 125 | 2000 | 2 |
| 62.5 | 1000 | 3 |
| 31.25 | 500 | 4 |
| 15.63 | 250 | 5 |
| 7.8 | 125 | 6 |
| 3.9 | 62.5 | 7 |
| 1.9 | 31.25 | 8 |
| 0.97 | 15.63 | 9 |
| 0.48 | 7.8 | 10 |
| Positive control (broth and microbial suspension) | Positive control (broth and microbial suspension) | 11 |
| Negative control (broth only) | Negative control (broth only) | 12 |

Positive control = broth plus microbial suspension; negative control = broth only. Each co-agent column applies to amlodipine, fluoxetine, escitalopram, or fluconazole.

**Table S7. Agar-well diffusion results for individual antimicrobial and non-antibiotic agents against clinical isolates of Staphylococcus aureus, Pseudomonas aeruginosa, and Candida albicans.**

| **Agent** | ***Staphylococcus aureus*** | | | | | ***Pseudomonas aeruginosa*** | | | | | ***Candida albicans*** | | | | | |
| --- | --- | --- | --- | --- | --- | --- | --- | --- | --- | --- | --- | --- | --- | --- | --- | --- |
|  | **Inhibition zone (mm)** | | | | **Image** | **Inhibition zone (mm)** | | | | **Image** | | **Inhibition zone (mm)** | | | | **Image** |
|  | **Run** | **Statistics** | | |  | **Run** | **Statistics** | | |  |  | **Run** | **Statistics** | | |  |
|  |  | **Zone** | **SD** | **Mean** |  |  | **Zone** | **SD** | **Mean** |  |  |  | **Zone** | **SD** | **Mean** |  |
| **Ciprofloxacin** | 1 | 45 | 1.41 | 46 |  | 1 | 42 | 4.24 | 39 |  | | 1 | 25 | 0.00 | 25 |  |
|  | 2 | 47 |  |  |  | 2 | 36 |  |  |  |  | 2 | 25 |  |  |  |
|  | NC | Pass |  |  |  | NC | Pass |  |  |  |  | NC | Pass |  |  |  |
| **Escitalopram** | 1 | 23 | 0.71 | 23.5 |  | 1 | 15 | 0.71 | 15.5 |  | | 1 | 30 | 0.00 | 30 |  |
|  | 2 | 24 |  |  |  | 2 | 16 |  |  |  |  | 2 | 30 |  |  |  |
|  | NC | Pass |  |  |  | NC | Pass |  |  |  |  | NC | Pass |  |  |  |
| **Fluconazole** | 1 | 19 | 0.00 | 19 |  | 1 | 0 | 0.00 | 0 |  | | 1 | 30 | 0.00 | 30 |  |
|  | 2 | 19 |  |  |  | 2 | 0 |  |  |  |  | 2 | 30 |  |  |  |
|  | NC | Pass |  |  |  | NC | Pass |  |  |  |  | NC | Pass |  |  |  |
| **Fluoxetine** | 1 | 41 | 1.41 | 40 |  | 1 | 26 | 1.41 | 27 |  | | 1 | 42 | 0.00 | 42 |  |
|  | 2 | 39 |  |  |  | 2 | 28 |  |  |  |  | 2 | 42 |  |  |  |
|  | NC | Pass |  |  |  | NC | Pass |  |  |  |  | NC | Pass |  |  |  |
| **Amlodipine** | 1 | 30 | 1.41 | 31 |  | 1 | 20 | 0.00 | 20 |  | | 1 | 38 | 4.95 | 41.5 |  |
|  | 2 | 32 |  |  |  | 2 | 20 |  |  |  |  | 2 | 45 |  |  |  |
|  | NC | Pass |  |  |  | NC | Pass |  |  |  |  | NC | Pass |  |  |  |

Values are raw inhibition-zone readings from two independent determinations; means and sample standard deviations were calculated from the two readings. Run 1 and Run 2 denote the two independent determinations. NC = negative control; Pass = the negative control produced no measurable inhibition. SD = standard deviation.

**Table S8. Agar-well diffusion results for individual antimicrobial and non-antibiotic agents against the reference strains Staphylococcus aureus ATCC 25923 and Pseudomonas aeruginosa ATCC 27853.**

| **Agent** | ***Staphylococcus aureus*** | | | | | ***Pseudomonas aeruginosa*** | | | | |  |
| --- | --- | --- | --- | --- | --- | --- | --- | --- | --- | --- | --- |
|  | **Inhibition zone (mm)** | | | | **Image** | **Inhibition zone (mm)** | | | | **Image** | |
|  | **Run** | **Statistics** | | |  | **Run** | **Statistics** | | |  |  |
|  |  | **Zone** | **SD** | **Mean** |  |  | **Zone** | **SD** | **Mean** |  |  |
| **Ciprofloxacin** | 1 | 53 | **1.41** | 52 |  | 1 | 63 | 0.71 | 62.5 |  | |
|  | 2 | 51 |  |  |  | 2 | 62 |  |  |  |  |
|  | NC | Pass |  |  |  | NC | Pass |  |  |  |  |
| **Escitalopram** | 1 | 30.9 | **1.70** | 29.7 |  | 1 | 39 | 0.71 | 39.5 |  | |
|  | 2 | 28.5 |  |  |  | 2 | 40 |  |  |  |  |
|  | NC | Pass |  |  |  | NC | Pass |  |  |  |  |
| **Fluconazole** | 1 | 23.8 | **0.57** | 23.4 |  | 1 | 24 | 0.42 | 24.3 |  | |
|  | 2 | 23 |  |  |  | 2 | 24.6 |  |  |  |  |
|  | NC | Pass |  |  |  | NC | Pass |  |  |  |  |
| **Fluoxetine** | 1 | 44 | **0.99** | 43.3 |  | 1 | 47 | 0.57 | 47.4 |  | |
|  | 2 | 42.6 |  |  |  | 2 | 47.8 |  |  |  |  |
|  | NC | Pass |  |  |  | NC | Pass |  |  |  |  |
| **Amlodipine** | 1 | 35 | **0.35** | 35.25 |  | 1 | 38.4 | 0.28 | 38.2 |  | |
|  | 2 | 35.5 |  |  |  | 2 | 38 |  |  |  |  |
|  | NC | Pass |  |  |  | NC | Pass |  |  |  |  |

Values are raw inhibition-zone readings from two independent determinations; means and sample standard deviations were calculated from the two readings. Run 1 and Run 2 denote the two independent determinations. NC = negative control; Pass = the negative control produced no measurable inhibition. ATCC = American Type Culture Collection; SD = standard deviation.

**Table S9. Agar-well diffusion worksheet for dual combinations against clinical isolates of Staphylococcus aureus, Pseudomonas aeruginosa, and Candida albicans.**

| **Combination** | ***Staphylococcus aureus*** | | | | | | | | ***Pseudomonas aeruginosa*** | | | | | | | |
| --- | --- | --- | --- | --- | --- | --- | --- | --- | --- | --- | --- | --- | --- | --- | --- | --- |
|  | **Component zones (mm)** | | | | | | | **Image** | **Component zones (mm)** | | | | | | | **Image** |
|  | **Run** | **Drug** | **Zone** | | | | |  | **Run** | **Drug** | **Zone** | | | | |  |
|  |  |  | **Zone** | **Mean** | | **Total SD** | **Total mean** |  |  |  | **Zone** | **Mean** | | **Total SD** | **Total mean** |  |
| **Ciprofloxacin, Escitalopram** | **1** | Cip | 46 | Cip | 43.5 | 2.83 | 71 |  | **1** | Cip | 48 | Cip | 46.5 | 4.24 | 68 |  |
|  |  | Esc | 27 |  |  |  |  |  |  | Esc | 23 |  |  |  |  |  |
|  |  | NC | Pass | Esc | 27.5 |  |  |  |  | NC | Pass | Esc | 21.5 |  |  |  |
|  | **2** | Cip | 41 |  |  |  |  |  | **2** | Cip | 45 |  |  |  |  |  |
|  |  | Esc | 28 | NC | 0 |  |  |  |  | Esc | 20 | NC | 0 |  |  |  |
|  |  | NC | Pass |  |  |  |  |  |  | NC | Pass |  |  |  |  |  |
| **Fluconazole, Ciprofloxacin** | **1** | FZ | 27 | FZ | 26 | 1.41 | 67 |  | **1** | FZ | 15 | FZ | 14 | 3.54 | 57.5 |  |
|  |  | Cip | 41 |  |  |  |  |  |  | Cip | 45 |  |  |  |  |  |
|  |  | NC | Pass | Cip | 41 |  |  |  |  | NC | Pass | Cip | 43.5 |  |  |  |
|  | **2** | FZ | 25 |  |  |  |  |  | **2** | FZ | 13 |  |  |  |  |  |
|  |  | Cip | 41 | NC | 0 |  |  |  |  | Cip | 42 | NC | 0 |  |  |  |
|  |  | NC | Pass |  |  |  |  |  |  | NC | Pass |  |  |  |  |  |
| **Amlodipine, Fluconazole** | **1** | Am | 40 | Am | 37.5 | 7.07 | 65 |  | **1** | Am | 30 | Am | 29 | 2.83 | 43 |  |
|  |  | FZ | 30 |  |  |  |  |  |  | FZ | 15 |  |  |  |  |  |
|  |  | NC | Pass | FZ | 27.5 |  |  |  |  | NC | Pass | FZ | 14 |  |  |  |
|  | **2** | Am | 35 |  |  |  |  |  | **2** | Am | 28 |  |  |  |  |  |
|  |  | FZ | 25 | NC | 0 |  |  |  |  | FZ | 13 | NC | 0 |  |  |  |
|  |  | NC | Pass |  |  |  |  |  |  | NC | Pass |  |  |  |  |  |
| **Fluconazole, Escitalopram** | **1** | FZ | 29 | FZ | 28 | 2.12 | 54.5 |  | **1** | FZ | 0 | FZ | 0 | 1.41 | 16 |  |
|  |  | Esc | 27 |  |  |  |  |  |  | Esc | 17 |  |  |  |  |  |
|  |  | NC | Pass | Esc | 26.5 |  |  |  |  | NC | Pass | Esc | 16 |  |  |  |
|  | **2** | FZ | 27 |  |  |  |  |  | **2** | FZ | 0 |  |  |  |  |  |
|  |  | Esc | 26 | NC | 0 |  |  |  |  | Esc | 15 | NC | 0 |  |  |  |
|  |  | NC | Pass |  |  |  |  |  |  | NC | Pass |  |  |  |  |  |
| **Amlodipine, Ciprofloxacin** | **1** | Am | 34 | Am | 34.5 | 0.00 | 75 |  | **1** | Am | 30 | Am | 31 | 2.12 | 78.5 |  |
|  |  | Cip | 41 |  |  |  |  |  |  | Cip | 47 |  |  | 2.12 |  |  |
|  |  | NC | Pass | Cip | 40.5 | 0.00 |  |  |  | NC | Pass | Cip | 47.5 |  |  |  |
|  | **2** | Am | 35 |  |  |  |  |  | **2** | Am | 32 |  |  |  |  |  |
|  |  | Cip | 40 | NC | 0 |  |  |  |  | Cip | 48 | NC | 0 |  |  |  |
|  |  | NC | Pass |  |  |  |  |  |  | NC | Pass |  |  |  |  |  |
| **Amlodipine, Escitalopram** | **1** | Am | 34 | Am | 34.5 | 2.83 | 61 |  | **1** | Am | 29 | Am | 29.5 | 0.71 | 45.5 |  |
|  |  | Esc | 25 |  |  |  |  |  |  | Esc | 17 |  |  |  |  |  |
|  |  | NC | Pass | Esc | 26.5 |  |  |  |  | NC | Pass | Esc | 16 |  |  |  |
|  | **2** | Am | 35 |  |  |  |  |  | **2** | Am | 30 |  |  |  |  |  |
|  |  | Esc | 28 | NC | 0 |  |  |  |  | Esc | 15 | NC | 0 |  |  |  |
|  |  | NC | Pass |  |  |  |  |  |  | NC | Pass |  |  |  |  |  |
| **Fluoxetine, Fluconazole** | **1** | FX | 49 | FX | 47 | 0.71 | 69.5 |  | **1** | FX | 27 | FX | 26.5 | 0.71 | 26.5 |  |
|  |  | FZ | 20 |  |  |  |  |  |  | FZ | 0 |  |  |  |  |  |
|  |  | NC | Pass | FZ | 22.5 |  |  |  |  | NC | Pass | FZ | 0 |  |  |  |
|  | **2** | FX | 45 |  |  |  |  |  | **2** | FX | 26 |  |  |  |  |  |
|  |  | FZ | 25 | NC | 0 |  |  |  |  | FZ | 0 | NC | 0 |  |  |  |
|  |  | NC | Pass |  |  |  |  |  |  | NC | Pass |  |  |  |  |  |
| **Fluoxetine, Escitalopram** | **1** | FX | 40 | FX | 41 | 0.00 | 70 |  | **1** | FX | 29 | FX | 28.5 | 2.12 | 42.5 |  |
|  |  | Esc | 30 |  |  |  |  |  |  | Esc | 15 |  |  |  |  |  |
|  |  | NC | Pass | Esc | 29 |  |  |  |  | NC | Pass | Esc | 14 |  |  |  |
|  | **2** | FX | 42 |  |  |  |  |  | **2** | FX | 28 |  |  |  |  |  |
|  |  | Esc | 28 | NC | 0 |  |  |  |  | Esc | 13 | NC | 0 |  |  |  |
|  |  | NC | Pass |  |  |  |  |  |  | NC | Pass |  |  |  |  |  |
| **Fluoxetine, Amlodipine** | **1** | FX | 40 | FX | 40 | 1.41 | 74 |  | **1** | FX | 26 | FX | 25.5 | 0.00 | 50 |  |
|  |  | Am | 33 |  |  |  |  |  |  | Am | 24 |  |  |  |  |  |
|  |  | NC | Pass | Am | 34 |  |  |  |  | NC | Pass | Am | 24.5 |  |  |  |
|  | **2** | FX | 40 |  |  |  |  |  | **2** | FX | 25 |  |  |  |  |  |
|  |  | Am | 35 | NC | 0 |  |  |  |  | Am | 25 | NC | 0 |  |  |  |
|  |  | NC | Pass |  |  |  |  |  |  | NC | Pass |  |  |  |  |  |
| **Fluoxetine, Ciprofloxacin** | **1** | FX | 41 | FX | 40.5 | 2.83 | 84 |  | **1** | FX | 28 | FX | 28.5 | 2.12 | 65.5 |  |
|  |  | Cip | 45 |  |  |  |  |  |  | Cip | 36 |  |  |  |  |  |
|  |  | NC | Pass | Cip | 43.5 | 2.83 |  |  |  | NC | Pass | Cip | 37 | 2.12 |  |  |
|  | **2** | FX | 40 |  |  |  |  |  | **2** | FX | 29 |  |  |  |  |  |
|  |  | Cip | 42 | NC | 0 |  |  |  |  | Cip | 38 | NC | 0 |  |  |  |
|  |  | NC | Pass |  |  |  |  |  |  | NC | Pass |  |  |  |  |  |

| **Combination** | ***Candida albicans*** | | | | | | | |  |
| --- | --- | --- | --- | --- | --- | --- | --- | --- | --- |
|  | **Component zones (mm)** | | | | | | | **Image** | |
|  | **Run** | **Drug** | **Zone** | | | | |  |  |
|  |  |  | **Zone** | **Mean** | | **Total SD** | **Total mean** |  |  |
| **Ciprofloxacin, Escitalopram** | **1** | Cip | 27 | Cip | 26.5 | 0.71 | 51.5 |  | |
|  |  | Esc | 25 |  |  |  |  |  |  |
|  |  | NC | Pass | Esc | 25 |  |  |  |  |
|  | **2** | Cip | 26 |  |  |  |  |  | |
|  |  | Esc | 25 | NC | 0 |  |  |  |  |
|  |  | NC | Pass |  |  |  |  |  |  |
| **Fluconazole, Ciprofloxacin** | **1** | FZ | 31 | FZ | 35.5 | 3.54 | 62.5 |  | |
|  |  | Cip | 29 |  |  |  |  |  |  |
|  |  | NC | Pass | Cip | 27 |  |  |  |  |
|  | **2** | FZ | 40 |  |  |  |  |  | |
|  |  | Cip | 25 | NC | 0 |  |  |  |  |
|  |  | NC | Pass |  |  |  |  |  |  |
| **Amlodipine, Fluconazole** | **1** | Am | 45 | Am | 45.5 | 0.00 | 90 |  | |
|  |  | FZ | 45 |  |  |  |  |  |  |
|  |  | NC | Pass | FZ | 44.5 |  |  |  |  |
|  | **2** | Am | 46 |  |  |  |  |  | |
|  |  | FZ | 44 | NC | 0 |  |  |  |  |
|  |  | NC | Pass |  |  | 0.00 |  |  |  |
| **Fluconazole, Escitalopram** | **1** | FZ | 45 | FZ | 44 | 1.41 | 44 |  | |
|  |  | Esc | 0 |  |  |  |  |  |  |
|  |  | NC | Pass | Esc | 0 |  |  |  |  |
|  | **2** | FZ | 43 |  |  |  |  |  | |
|  |  | Esc | 0 | NC | 0 |  |  |  |  |
|  |  | NC | Pass |  |  |  |  |  |  |
| **Amlodipine, Ciprofloxacin** | **1** | Am | 32 | Am | 33.5 | 0.71 | 62.5 |  | |
|  |  | Cip | 30 |  |  |  |  |  |  |
|  |  | NC | Pass | Cip | 29 |  |  |  |  |
|  | **2** | Am | 35 |  |  |  |  |  | |
|  |  | Cip | 28 | NC | 0 |  |  |  |  |
|  |  | NC | Pass |  |  |  |  |  |  |
| **Amlodipine, Escitalopram** | **1** | Am | 35 | Am | 34 | 4.95 | 56.5 |  | |
|  |  | Esc | 25 |  |  |  |  |  |  |
|  |  | NC | Pass | Esc | 22.5 |  |  |  |  |
|  | **2** | Am | 33 |  |  |  |  |  | |
|  |  | Esc | 20 | NC | 0 |  |  |  |  |
|  |  | NC | Pass |  |  |  |  |  |  |
| **Fluoxetine, Fluconazole** | **1** | FX | 51 | FX | 50.5 | 3.54 | 96.5 |  | |
|  |  | FZ | 48 |  |  |  |  |  |  |
|  |  | NC | Pass | FZ | 46 |  |  |  |  |
|  | **2** | FX | 50 |  |  |  |  |  | |
|  |  | FZ | 44 | NC | 0 |  |  |  |  |
|  |  | NC | Pass |  |  |  |  |  |  |
| **Fluoxetine, Escitalopram** | **1** | FX | 46 | FX | 46.5 | 0.00 | 87 |  | |
|  |  | Esc | 41 |  |  |  |  |  |  |
|  |  | NC | Pass | Esc | 40.5 |  |  |  |  |
|  | **2** | FX | 47 |  |  |  |  |  | |
|  |  | Esc | 40 | NC | 0 |  |  |  |  |
|  |  | NC | Pass |  |  |  |  |  |  |
| **Fluoxetine, Amlodipine** | **1** | FX | 42 | FX | 43.5 | 3.54 | 78.5 |  | |
|  |  | Am | 34 |  |  |  |  |  |  |
|  |  | NC | Pass | Am | 35 |  |  |  |  |
|  | **2** | FX | 45 |  |  |  |  |  | |
|  |  | Am | 36 | NC | 0 |  |  |  |  |
|  |  | NC | Pass |  |  |  |  |  |  |
| **Fluoxetine, Ciprofloxacin** | **1** | FX | 45 | FX | 44 | 0.00 | 67 |  | |
|  |  | Cip | 22 |  |  |  |  |  |  |
|  |  | NC | Pass | Cip | 23 |  |  |  |  |
|  | **2** | FX | 43 |  |  |  |  |  | |
|  |  | Cip | 24 | NC | 0 |  |  |  |  |
|  |  | NC | Pass |  |  |  |  |  |  |

Component-level readings and aggregate totals are retained as laboratory worksheet records. Aggregate means and sample standard deviations were calculated from the two determination-level totals. These diffusion-derived values are screening outcomes and were not used to define synergy. Run 1 and Run 2 denote the two independent determinations. NC = negative control; Cip = ciprofloxacin; Esc = escitalopram; FX = fluoxetine; FZ = fluconazole; Am = amlodipine.

**Table S10. Agar-well diffusion worksheet for triplet combinations against clinical isolates of Staphylococcus aureus, Pseudomonas aeruginosa, and Candida albicans.**

| **Combination** | ***Staphylococcus aureus*** | | | | | | | | ***Pseudomonas aeruginosa*** | | | | | | | |
| --- | --- | --- | --- | --- | --- | --- | --- | --- | --- | --- | --- | --- | --- | --- | --- | --- |
|  | **Component zones (mm)** | | | | | | | **Image** | **Component zones (mm)** | | | | | | | **Image** |
|  | **Run** | **Drug** | **Zone** | | | | |  | **Run** | **Drug** | **Zone** | | | | |  |
|  |  |  | **Zone** | **Mean** | | **SD** | **Mean** |  |  |  | **Zone** | **Mean** | | **SD** | **Mean** |  |
| **Ciprofloxacin, Fluoxetine, Fluconazole** | **1** | Cip | 46 | Cip | 44 | 2.8 | 99 |  | **1** | Cip | 38 | Cip | 37 | 2.1 | 58.5 |  |
|  |  | FX | 30 |  |  |  |  |  |  | FX | 22 |  |  |  |  |  |
|  |  | FZ | 25 | FX | 30.5 |  |  |  |  | FZ | 0 | FX | 21.5 |  |  |  |
|  |  | NC | Pass |  |  |  |  |  |  | NC | Pass |  |  |  |  |  |
|  | **2** | Cip | 42 | FZ | 24.5 |  |  |  | **2** | Cip | 36 | FZ | 0 |  |  |  |
|  |  | FX | 31 |  |  |  |  |  |  | FX | 21 |  |  |  |  |  |
|  |  | FZ | 24 | NC | 0 |  |  |  |  | FZ | 0 | NC | 0 |  |  |  |
|  |  | NC | Pass |  |  |  |  |  |  | NC | Pass |  |  |  |  |  |
| **Ciprofloxacin, Fluoxetine, Amlodipine** | **1** | Cip | 40 | Cip | 40.5 | 2.8 | 101 |  | **1** | Cip | 36 | Cip | 37 | 2.8 | 80 |  |
|  |  | FX | 30 |  |  |  |  |  |  | FX | 23 |  |  |  |  |  |
|  |  | Am | 32 | FX | 31 |  |  |  |  | Am | 23 | FX | 21.5 |  |  |  |
|  |  | NC | Pass |  |  |  |  |  |  | NC | Pass |  |  |  |  |  |
|  | **2** | Cip | 41 | Am | 29.5 |  |  |  | **2** | Cip | 38 | Am | 21.5 |  | 80 |  |
|  |  | FX | 32 |  |  |  |  |  |  | FX | 20 |  |  |  |  |  |
|  |  | Am | 27 | NC | 0 |  |  |  |  | Am | 20 | NC | 0 |  |  |  |
|  |  | NC | Pass |  |  |  |  |  |  | NC | Pass |  |  |  |  |  |
| **Ciprofloxacin, Fluoxetine, Escitalopram** | **1** | Cip | 36 | Cip | 38 | 5.7 | 92 |  | **1** | Cip | 40 | Cip | 42.5 | 3.5 | 88.5 |  |
|  |  | FX | 30 |  |  |  |  |  |  | FX | 30 |  |  |  |  |  |
|  |  | Esc | 22 | FX | 30 |  |  |  |  | Esc | 16 | FX | 31 |  |  |  |
|  |  | NC | Pass |  |  |  |  |  |  | NC | Pass |  |  |  |  |  |
|  | **2** | Cip | 40 | Esc | 24 |  |  |  | **2** | Cip | 45 | Esc | 15 |  |  |  |
|  |  | FX | 30 |  |  |  |  |  |  | FX | 32 |  |  |  |  |  |
|  |  | Esc | 26 | NC | 0 |  |  |  |  | Esc | 14 | NC | 0 |  |  |  |
|  |  | NC | Pass |  |  |  |  |  |  | NC | Pass |  |  |  |  |  |
| **Ciprofloxacin, Amlodipine, Fluconazole** | **1** | Cip | 44 | Cip | 44 | 3.5 | 95.5 |  | **1** | Cip | 40 | Cip | 40 | 1.4 | 66 |  |
|  |  | Am | 27 |  |  |  |  |  |  | Am | 25 |  |  |  |  |  |
|  |  | FZ | 27 | Am | 26 |  |  |  |  | FZ | 0 | Am | 26 |  |  |  |
|  |  | NC | Pass |  |  |  |  |  |  | NC | Pass |  |  |  |  |  |
|  | **2** | Cip | 44 | FZ | 25.5 |  |  |  | **2** | Cip | 40 | FZ | 0 |  |  |  |
|  |  | Am | 25 |  |  |  |  |  |  | Am | 27 |  |  |  |  |  |
|  |  | FZ | 24 | NC | 0 |  |  |  |  | FZ | 0 | NC | 0 |  |  |  |
|  |  | NC | Pass |  |  |  |  |  |  | NC | Pass |  |  |  |  |  |
| **Ciprofloxacin, Escitalopram, Fluconazole** | **1** | Cip | 30 | Cip | 30 | 4.2 | 74 |  | **1** | Cip | 37 | Cip | 37 | 0.0 | 52 |  |
|  |  | Esc | 22 |  |  |  |  |  |  | Esc | 15 |  |  |  |  |  |
|  |  | FZ | 25 | Esc | 21.5 |  |  |  |  | FZ | 0 | Esc | 15 |  |  |  |
|  |  | NC | Pass |  |  |  |  |  |  | NC | Pass |  |  |  |  |  |
|  | **2** | Cip | 30 | FZ | 22.5 |  |  |  | **2** | Cip | 37 | FZ | 0 |  |  |  |
|  |  | Esc | 21 |  |  |  |  |  |  | Esc | 15 |  |  |  |  |  |
|  |  | FZ | 20 | NC | 0 |  |  |  |  | FZ | 0 | NC | 0 |  |  |  |
|  |  | NC | Pass |  |  |  |  |  |  | NC | Pass |  |  |  |  |  |
| **Ciprofloxacin, Escitalopram, Amlodipine** | **1** | Cip | 40 | Cip | 39.5 | 1.4 | 90 |  | **1** | Cip | 40 | Cip | 39 | 3.5 | 77.5 |  |
|  |  | Esc | 21 |  |  |  |  |  |  | Esc | 16 |  |  |  |  |  |
|  |  | Am | 30 | Esc | 21 |  |  |  |  | Am | 24 | Esc | 14.5 |  |  |  |
|  |  | NC | Pass |  |  |  |  |  |  | NC | Pass |  |  |  |  |  |
|  | **2** | Cip | 39 | Am | 29.5 |  |  |  | **2** | Cip | 38 | Am | 24 |  |  |  |
|  |  | Esc | 21 |  |  |  |  |  |  | Esc | 13 |  |  |  |  |  |
|  |  | Am | 29 | NC | 0 |  |  |  |  | Am | 24 | NC | 0 |  |  |  |
|  |  | NC | Pass |  |  |  |  |  |  | NC | Pass |  |  |  |  |  |
| **Amlodipine, Fluoxetine, Fluconazole** | **1** | Am | 30 | Am | 30 | 2.1 | 86.5 |  | **1** | Am | 24 | Am | 23 | 1.4 | 47 |  |
|  |  | FX | 31 |  |  |  |  |  |  | FX | 24 |  |  |  |  |  |
|  |  | FZ | 24 | FX | 32 |  |  |  |  | FZ | 0 | FX | 24 |  |  |  |
|  |  | NC | Pass |  |  |  |  |  |  | NC | Pass |  |  |  |  |  |
|  | **2** | Am | 30 | FZ | 24.5 | 2.1 |  |  | **2** | Am | 22 | FZ | 0 | 1.4 |  |  |
|  |  | FX | 33 |  |  |  |  |  |  | FX | 24 |  |  |  |  |  |
|  |  | FZ | 25 | NC | 0 |  |  |  |  | FZ | 0 | NC | 0 |  |  |  |
|  |  | NC | Pass |  |  |  |  |  |  | NC | Pass |  |  |  |  |  |
| **Amlodipine, Escitalopram, Fluconazole** | **1** | Am | 25 | Am | 25 | 0.7 | 76.5 |  | **1** | Am | 25 | Am | 24.5 | 1.4 | 38 |  |
|  |  | Esc | 25 |  |  |  |  |  |  | Esc | 14 |  |  |  |  |  |
|  |  | FZ | 27 | Esc | 24 |  |  |  |  | FZ | 0 | Esc | 13.5 |  |  |  |
|  |  | NC | Pass |  |  |  |  |  |  | NC | Pass |  |  |  |  |  |
|  | **2** | Am | 25 | FZ | 27.5 |  |  |  | **2** | Am | 24 | FZ | 0 |  |  |  |
|  |  | Esc | 23 |  |  |  |  |  |  | Esc | 13 |  |  |  |  |  |
|  |  | FZ | 28 | NC | 0 |  |  |  |  | FZ | 0 | NC | 0 |  |  |  |
|  |  | NC | Pass |  |  |  |  |  |  | NC | Pass |  |  |  |  |  |
| **Amlodipine, Escitalopram, Fluoxetine** | **1** | Am | 29 | Am | 29 | 0.7 | 82.5 |  | **1** | Am | 25 | Am | 25.5 | 1.4 | 62 |  |
|  |  | Esc | 24 |  |  |  |  |  |  | Esc | 14 |  |  |  |  |  |
|  |  | FX | 30 | Esc | 23.5 |  |  |  |  | FX | 22 | Esc | 15 |  |  |  |
|  |  | NC | Pass |  |  |  |  |  |  | NC | Pass |  |  |  |  |  |
|  | **2** | Am | 29 | FX | 30 |  |  |  | **2** | Am | 26 | FX | 21.5 |  |  |  |
|  |  | Esc | 23 |  |  |  |  |  |  | Esc | 16 |  |  |  |  |  |
|  |  | FX | 30 | NC | 0 |  |  |  |  | FX | 21 | NC | 0 |  |  |  |
|  |  | NC | Pass |  |  |  |  |  |  | NC | Pass |  |  |  |  |  |
| **Escitalopram, Fluoxetine, Fluconazole** | **1** | Esc | 23 | Esc | 22.5 | 0.0 | 76 |  | **1** | Esc | 16 | Esc | 16 | Not determined | Not determined |  |
|  |  | FX | 25 |  |  |  |  |  |  | FX | 22 |  |  |  |  |  |
|  |  | FZ | 28 | FX | 23.5 |  |  |  |  | FZ | 0 | FX | 22 |  |  |  |
|  |  | NC | Pass |  |  |  |  |  |  | NC | Pass |  |  |  |  |  |
|  | **2** | Esc | 22 | FZ | 30 |  |  |  | **2** | Esc |  | FZ | 0 |  |  |  |
|  |  | FX | 22 |  |  |  |  |  |  | FX |  |  |  |  |  |  |
|  |  | FZ | 32 | NC | 0 |  |  |  |  | FZ |  | NC | 0 |  |  |  |
|  |  | NC | Pass |  |  |  |  |  |  | NC | Pass |  |  |  |  |  |

| **Combination** | ***Candida albicans*** | | | | | | | | | |
| --- | --- | --- | --- | --- | --- | --- | --- | --- | --- | --- |
|  | **Component zones (mm)** | | | | | | | | **Status** | **Image** |
|  | **Run** | **Drug** | **Zone** | | **Result** | | | |  |  |
|  |  |  | **Zone** | **Mean** | | | **SD** | **Mean** |  |  |
| **Ciprofloxacin, Fluoxetine, Fluconazole** | **1** | Cip | 24 | Cip | | 24.5 | 0.7 | 126.5 | Screening only |  |
|  |  | FX | 38 |  |  |  |  |  |  |  |
|  |  | FZ | 64 | FX | | 37.5 |  |  |  |  |
|  |  | NC | Pass |  |  |  |  |  |  |  |
|  | **2** | Cip | 25 | FZ | | 64.5 |  |  | Screening only |  |
|  |  | FX | 37 |  |  |  |  |  |  |  |
|  |  | FZ | 65 | NC | | 0 |  |  |  |  |
|  |  | NC | Pass |  |  |  |  |  |  |  |
| **Ciprofloxacin, Fluoxetine, Amlodipine** | **1** | Cip | 28 | Cip | | 27.5 | 1.4 | 107 | Screening only |  |
|  |  | FX | 32 |  |  |  |  |  |  |  |
|  |  | Am | 48 | FX | | 32.5 |  |  |  |  |
|  |  | NC | Pass |  |  |  |  |  |  |  |
|  | **2** | Cip | 27 | Am | | 47 |  |  | Screening only |  |
|  |  | FX | 33 |  |  |  |  |  |  |  |
|  |  | Am | 46 | NC | | 0 |  |  |  |  |
|  |  | NC | Pass |  |  |  |  |  |  |  |
| **Ciprofloxacin, Fluoxetine, Escitalopram** | **1** | Cip | 22 | Cip | | 23.5 | 2.8 | 87 | Screening only |  |
|  |  | FX | 40 |  |  |  |  |  |  |  |
|  |  | Esc | 23 | FX | | 41 |  |  |  |  |
|  |  | NC | Pass |  |  |  |  |  |  |  |
|  | **2** | Cip | 25 | Esc | | 22.5 |  |  | Screening only |  |
|  |  | FX | 42 |  |  |  |  |  |  |  |
|  |  | Esc | 22 | NC | | 0 |  |  |  |  |
|  |  | NC | Pass |  |  |  |  |  |  |  |
| **Ciprofloxacin, Amlodipine, Fluconazole** | **1** | Cip | 30 | Cip | | 29.5 | 2.1 | 138.5 | Screening only |  |
|  |  | Am | 45 |  |  |  |  |  |  |  |
|  |  | FZ | 62 | Am | | 46 |  |  |  |  |
|  |  | NC | Pass |  |  |  | 2.1 |  |  |  |
|  | **2** | Cip | 29 | FZ | | 63 |  |  | Screening only |  |
|  |  | Am | 47 |  |  |  |  |  |  |  |
|  |  | FZ | 64 | NC | | 0 |  |  |  |  |
|  |  | NC | Pass |  |  |  |  |  |  |  |
| **Ciprofloxacin, Escitalopram, Fluconazole** | **1** | Cip | 25 | Cip | | 27.5 | 0.7 | 97.5 | Screening only |  |
|  |  | Esc | 30 |  |  |  |  |  |  |  |
|  |  | FZ | 42 | Esc | | 27.5 |  |  |  |  |
|  |  | NC | Pass |  |  |  |  |  |  |  |
|  | **2** | Cip | 30 | FZ | | 42.5 |  |  | Screening only |  |
|  |  | Esc | 25 |  |  |  |  |  |  |  |
|  |  | FZ | 43 | NC | | 0 |  |  |  |  |
|  |  | NC | Pass |  |  |  |  |  |  |  |
| **Ciprofloxacin, Escitalopram, Amlodipine** | **1** | Cip | 29 | Cip | | 28 | 2.1 | 91.5 | Screening only |  |
|  |  | Esc | 26 |  |  |  |  |  |  |  |
|  |  | Am | 38 | Esc | | 25.5 |  |  |  |  |
|  |  | NC | Pass |  |  |  |  |  |  |  |
|  | **2** | Cip | 27 | Am | | 38 |  |  | Screening only |  |
|  |  | Esc | 25 |  |  |  |  |  |  |  |
|  |  | Am | 38 | NC | | 0 |  |  |  |  |
|  |  | NC | Pass |  |  |  |  |  |  |  |
| **Amlodipine, Fluoxetine, Fluconazole** | **1** | Am | 41 | Am | | 40 | 0.0 | 134 | Screening only |  |
|  |  | FX | 43 |  |  |  |  |  |  |  |
|  |  | FZ | 50 | FX | | 43 |  |  |  |  |
|  |  | NC | Pass |  |  |  |  |  |  |  |
|  | **2** | Am | 39 | FZ | | 51 |  |  | Screening only |  |
|  |  | FX | 43 |  |  |  |  |  |  |  |
|  |  | FZ | 52 | NC | | 0 |  |  |  |  |
|  |  | NC | Pass |  |  |  |  |  |  |  |
| **Amlodipine, Escitalopram, Fluconazole** | **1** | Am | 42 | Am | | 40.5 | 0.7 | 116.5 | Screening only |  |
|  |  | Esc | 24 |  |  |  |  |  |  |  |
|  |  | FZ | 50 | Esc | | 24.5 |  |  |  |  |
|  |  | NC | Pass |  |  |  |  |  |  |  |
|  | **2** | Am | 39 | FZ | | 51.5 |  |  | Screening only |  |
|  |  | Esc | 25 |  |  |  |  |  |  |  |
|  |  | FZ | 53 | NC | | 0 |  |  |  |  |
|  |  | NC | Pass |  |  |  |  |  |  |  |
| **Amlodipine, Escitalopram, Fluoxetine** | **1** | Am | 40 | Am | | 39 | 0.7 | 98.5 | Screening only |  |
|  |  | Esc | 25 |  |  |  |  |  |  |  |
|  |  | FX | 33 | Esc | | 25.5 |  |  |  |  |
|  |  | NC | Pass |  |  |  |  |  |  |  |
|  | **2** | Am | 38 | FX | | 34 |  |  | Screening only |  |
|  |  | Esc | 26 |  |  |  |  |  |  |  |
|  |  | FX | 35 | NC | | 0 |  |  |  |  |
|  |  | NC | Pass |  |  |  |  |  |  |  |

Table S10 (continued).

| **Combination** | ***Candida albicans*** | | | | | | | | | |
| --- | --- | --- | --- | --- | --- | --- | --- | --- | --- | --- |
|  | **Component zones (mm)** | | | | | | | | **Status** | **Image** |
|  | **Run** | **Drug** | **Zone** | | **Result** | | | |  |  |
|  |  |  | **Zone** | **Mean** | | | **SD** | **Mean** |  |  |
| **Escitalopram, Fluoxetine, Fluconazole** | **1** | Esc | 26 | Esc | | 25.5 | 0.7 | 105.5 | Screening only |  |
|  |  | FX | 38 |  |  |  |  |  |  |  |
|  |  | FZ | 42 | FX | | 38 |  |  |  |  |
|  |  | NC | Pass |  |  |  |  |  |  |  |
|  | **2** | Esc | 25 | FZ | | 42 |  |  |  |  |
|  |  | FX | 38 |  |  |  |  |  |  |  |
|  |  | FZ | 42 | NC | | 0 |  |  |  |  |
|  |  | NC | Pass |  |  |  |  |  |  |  |

For the triplet combinations also reported in Table 3 of the main manuscript, the mean inhibition zones and standard deviations shown here match the corresponding main-table values. Component-level readings are retained as laboratory worksheet records. Diffusion results are screening outcomes and do not define synergy; formal interaction classification is based on the checkerboard fractional inhibitory concentration index (FICI). Run 1 and Run 2 denote the two independent determinations. NC = negative control; Cip = ciprofloxacin; Esc = escitalopram; FX = fluoxetine; FZ = fluconazole; Am = amlodipine. Not determined indicates incomplete replicate data.

**Table S11. Agar-well diffusion worksheet for triplet combinations against the reference strains Staphylococcus aureus ATCC 25923 and Pseudomonas aeruginosa ATCC 27853.**

| **Combination** | ***Staphylococcus aureus ATCC 25923*** | | | | | | | | | ***Pseudomonas aeruginosa ATCC 27853*** | | | | | | | | | | |
| --- | --- | --- | --- | --- | --- | --- | --- | --- | --- | --- | --- | --- | --- | --- | --- | --- | --- | --- | --- | --- |
|  | **Component zones (mm)** | | | | | | | **Status** | **Image** | **Component zones (mm)** | | | | | | | | **Status** | | **Image** |
|  | **Run** | **Drug** | **Zone** | | | | |  |  | **Run** | **Drug** | **Zone** | | | | | | **Status** | | Images |
|  |  |  | **Zone** | **Mean** | | **Total SD** | **Total mean** |  |  |  |  | **Zone** | **Mean** | | **Total SD** | **Total mean** |  | |  | |
| **Ciprofloxacin, Fluoxetine, Fluconazole** | **1** | Cip | 51 | Cip | 51 | 1.4 | 108 | Screening only |  | **1** | Cip | 60 | Cip | 60 | 4.2 | 129 | Screening only | |  | |
|  |  | FX | 38 |  |  |  |  |  |  |  | FX | 42 |  |  |  |  |  |  |  |  |
|  |  | FZ | 20 | FX | 37 |  |  |  |  |  | FZ | 30 | FX | 42 |  |  |  |  |  |  |
|  |  | NC | Pass |  |  |  |  |  |  |  | NC | Pass |  |  |  |  |  |  |  |  |
|  | **2** | Cip | 51 | FZ | 20 |  |  |  |  | **2** | Cip | 60 | FZ | 27 |  |  |  |  |  | |
|  |  | FX | 36 |  |  |  |  |  |  |  | FX | 42 |  |  |  |  |  |  |  |  |
|  |  | FZ | 20 | NC | 0 |  |  |  |  |  | FZ | 24 | NC | 0 |  |  |  |  |  |  |
|  |  | NC | Pass |  |  |  |  |  |  |  | NC | Pass |  |  |  |  |  |  |  |  |
| **Ciprofloxacin, Fluoxetine, Amlodipine** | **1** | Cip | 50 | Cip | 49.5 | 0.0 | 117 | Screening only |  | **1** | Cip | 64 | Cip | 65 | 1.4 | 143 | Screening only | |  | |
|  |  | FX | 37 |  |  |  |  |  |  |  | FX | 48 |  |  |  |  |  |  |  |  |
|  |  | Am | 30 | FX | 37.5 |  |  |  |  |  | Am | 32 | FX | 46 |  |  |  |  |  |  |
|  |  | NC | Pass |  |  |  |  |  |  |  | NC | Pass |  |  |  |  |  |  |  |  |
|  | **2** | Cip | 49 | Am | 30 |  |  |  |  | **2** | Cip | 66 | Am | 32 |  |  |  |  |  | |
|  |  | FX | 38 |  |  |  |  |  |  |  | FX | 44 |  |  |  |  |  |  |  |  |
|  |  | Am | 30 | NC | 0 |  |  |  |  |  | Am | 32 | NC | 0 |  |  |  |  |  |  |
|  |  | NC | Pass |  |  |  |  |  |  |  | NC | Pass |  |  |  |  |  |  |  |  |
| **Ciprofloxacin, Fluoxetine, Escitalopram** | **1** | Cip | 48 | Cip | 49 | 1.4 | 119 | Screening only |  | **1** | Cip | 62 | Cip | 60 | 2.8 | 139 | Screening only | |  | |
|  |  | FX | 40 |  |  |  |  |  |  |  | FX | 41 |  |  |  |  |  |  |  |  |
|  |  | Esc | 30 | FX | 40 | 1.4 |  |  |  |  | Esc | 38 | FX | 41.5 |  |  |  |  |  |  |
|  |  | NC | Pass |  |  |  |  |  |  |  | NC | Pass |  |  |  |  |  |  |  |  |
|  | **2** | Cip | 50 | Esc | 30 |  |  |  |  | **2** | Cip | 58 | Esc | 37.5 |  |  |  |  |  | |
|  |  | FX | 40 |  |  |  |  |  |  |  | FX | 42 |  |  |  |  |  |  |  |  |
|  |  | Esc | 30 | NC | 0 |  |  |  |  |  | Esc | 37 | NC | 0 |  |  |  |  |  |  |
|  |  | NC | Pass |  |  |  |  |  |  |  | NC | Pass |  |  |  |  |  |  |  |  |
| **Ciprofloxacin, Amlodipine, Fluconazole** | **1** | Cip | 47 | Cip | 47.5 | 0.7 | 98.5 | Screening only |  | **1** | Cip | 62 | Cip | 60 | 2.1 | 128.5 | Screening only | |  | |
|  |  | Am | 32 |  |  |  |  |  |  |  | Am | 40 |  |  |  |  |  |  |  |  |
|  |  | FZ | 19 | Am | 31.5 |  |  |  |  |  | FZ | 28 | Am | 42.5 |  |  |  |  |  |  |
|  |  | NC | Pass |  |  |  |  |  |  |  | NC | Pass |  |  |  |  |  |  |  |  |
|  | **2** | Cip | 48 | FZ | 19.5 |  |  |  |  | **2** | Cip | 58 | FZ | 26 |  |  |  |  |  | |
|  |  | Am | 31 |  |  |  |  |  |  |  | Am | 45 |  |  |  |  |  |  |  |  |
|  |  | FZ | 20 | NC | 0 |  |  |  |  |  | FZ | 24 | NC | 0 |  |  |  |  |  |  |
|  |  | NC | Pass |  |  |  |  |  |  |  | NC | Pass |  |  |  |  |  |  |  |  |
| **Ciprofloxacin, Escitalopram, Fluconazole** | **1** | Cip | 48 | Cip | 48 | 0.0 | 98 | Screening only |  | **1** | Cip | 60 | Cip | 59 | 1.4 | 125 | Screening only | |  | |
|  |  | Esc | 32 |  |  |  |  |  |  |  | Esc | 40 |  |  |  |  |  |  |  |  |
|  |  | FZ | 18 | Esc | 31.5 |  |  |  |  |  | FZ | 26 | Esc | 39 |  |  |  |  |  |  |
|  |  | NC | Pass |  |  |  |  |  |  |  | NC | Pass |  |  |  |  |  |  |  |  |
|  | **2** | Cip | 48 | FZ | 18.5 |  |  |  |  | **2** | Cip | 58 | FZ | 27 |  |  |  |  |  | |
|  |  | Esc | 31 |  |  |  |  |  |  |  | Esc | 38 |  |  |  |  |  |  |  |  |
|  |  | FZ | 19 | NC | 0 |  | 98 |  |  |  | FZ | 28 | NC | 0 |  | 125 |  |  |  |  |
|  |  | NC | Pass |  |  |  |  |  |  |  | NC | Pass |  |  |  |  |  |  |  |  |
| **Ciprofloxacin, Escitalopram, Amlodipine** | **1** | Cip | 46 | Cip | 46.5 | 0.7 | 109.5 | Screening only |  | **1** | Cip | 66 | Cip | 64 | 7.1 | 135 | Screening only | |  | |
|  |  | Esc | 31 |  |  |  |  |  |  |  | Esc | 40 |  |  |  |  |  |  |  |  |
|  |  | Am | 32 | Esc | 31.5 | 0.7 |  |  |  |  | Am | 34 | Esc | 38 |  |  |  |  |  |  |
|  |  | NC | Pass |  |  |  |  |  |  |  | NC | Pass |  |  |  |  |  |  |  |  |
|  | **2** | Cip | 47 | Am | 31.5 |  |  |  |  | **2** | Cip | 62 | Am | 33 |  |  |  |  |  | |
|  |  | Esc | 32 |  |  |  |  |  |  |  | Esc | 36 |  |  |  |  |  |  |  |  |
|  |  | Am | 31 | NC | 0 |  |  |  |  |  | Am | 32 | NC | 0 |  |  |  |  |  |  |
|  |  | NC | Pass |  |  |  |  |  |  |  | NC | Pass |  |  |  |  |  |  |  |  |
| **Amlodipine, Fluoxetine, Fluconazole** | **1** | Am | 32 | Am | 31.5 | 1.4 | 90 | Screening only |  | **1** | Am | 35 | Am | 36.5 | 0.7 | 108.5 | Screening only | |  | |
|  |  | FX | 36 |  |  |  |  |  |  |  | FX | 44 |  |  |  |  |  |  |  |  |
|  |  | FZ | 21 | FX | 38 |  |  |  |  |  | FZ | 30 | FX | 43 |  |  |  |  |  |  |
|  |  | NC | Pass |  |  |  |  |  |  |  | NC | Pass |  |  |  |  |  |  |  |  |
|  | **2** | Am | 31 | FZ | 20.5 |  |  |  |  | **2** | Am | 38 | FZ | 29 |  |  |  |  |  | |
|  |  | FX | 40 |  |  |  |  |  |  |  | FX | 42 |  |  |  |  |  |  |  |  |
|  |  | FZ | 20 | NC | 0 |  |  |  |  |  | FZ | 28 | NC | 0 |  |  |  |  |  |  |
|  |  | NC | Pass |  |  |  |  |  |  |  | NC | Pass |  |  |  |  |  |  |  |  |
| **Amlodipine, Escitalopram, Fluconazole** | **1** | Am | 28 | Am | 28.5 | 2.1 | 78.5 | Screening only |  | **1** | Am | 38 | Am | 37 | 5.7 | 101 | Screening only | |  | |
|  |  | Esc | 30 |  |  |  |  |  |  |  | Esc | 40 |  |  |  |  |  |  |  |  |
|  |  | FZ | 19 | Esc | 30.5 |  |  |  |  |  | FZ | 27 | Esc | 39 |  |  |  |  |  |  |
|  |  | NC | Pass |  |  |  |  |  |  |  | NC | Pass |  |  |  |  |  |  |  |  |
|  | **2** | Am | 29 | FZ | 19.5 |  |  |  |  | **2** | Am | 36 | FZ | 25 |  |  |  |  |  | |
|  |  | Esc | 31 |  |  |  |  |  |  |  | Esc | 38 |  |  |  |  |  |  |  |  |
|  |  | FZ | 20 | NC | 0 |  |  |  |  |  | FZ | 23 | NC | 0 |  |  |  |  |  |  |
|  |  | NC | Pass |  |  |  |  |  |  |  | NC | Pass |  |  |  |  |  |  |  |  |
| **Amlodipine, Escitalopram, Fluoxetine** | **1** | Am | 32 | Am | 31.5 | 1.4 | 98 | Screening only |  | **1** | Am | 34 | Am | 34 | 0.7 | 111.5 | Screening only | |  | |
|  |  | Esc | 33 |  |  |  |  |  |  |  | Esc | 36 |  |  |  |  |  |  |  |  |
|  |  | FX | 34 | Esc | 32.5 | 1.4 |  |  |  |  | FX | 41 | Esc | 36 |  |  |  |  |  |  |
|  |  | NC | Pass |  |  |  |  |  |  |  | NC | Pass |  |  |  |  |  |  |  |  |
|  | **2** | Am | 31 | FX | 34 |  |  |  |  | **2** | Am | 34 | FX | 41.5 |  |  |  |  |  | |
|  |  | Esc | 32 |  |  |  |  |  |  |  | Esc | 36 |  |  |  |  |  |  |  |  |
|  |  | FX | 34 | NC | 0 |  |  |  |  |  | FX | 42 | NC | 0 |  |  |  |  |  |  |
|  |  | NC | Pass |  |  |  |  |  |  |  | NC | Pass |  |  |  |  |  |  |  |  |
| **Escitalopram, Fluoxetine, Fluconazole** | **1** | Esc | 35 | Esc | 35.5 | 1.4 | 88 | Screening only |  | **1** | Esc | 40 | Esc | 39 | 0.7 | 102.5 | Screening only | |  | |
|  |  | FX | 32 |  |  |  |  |  |  |  | FX | 40 |  |  |  |  |  |  |  |  |
|  |  | FZ | 20 | FX | 32 |  |  |  |  |  | FZ | 23 | FX | 40 |  |  |  |  |  |  |
|  |  | NC | Pass |  |  |  |  |  |  |  | NC | Pass |  |  |  |  |  |  |  |  |
|  | **2** | Esc | 36 | FZ | 20.5 |  |  |  |  | **2** | Esc | 38 | FZ | 23.5 |  |  |  |  |  | |
|  |  | FX | 32 |  |  |  |  |  |  |  | FX | 40 |  |  |  |  |  |  |  |  |
|  |  | FZ | 21 | NC | 0 |  |  |  |  |  | FZ | 24 | NC | 0 |  |  |  |  |  |  |
|  |  | NC | Pass |  |  |  |  |  |  |  | NC | Pass |  |  |  |  |  |  |  |  |

Component-level readings and aggregate totals are retained as laboratory worksheet records. Aggregate means and sample standard deviations were calculated from the two determination-level totals. Diffusion results are screening outcomes and do not define synergy. Run 1 and Run 2 denote the two independent determinations. NC = negative control; ATCC = American Type Culture Collection; Cip = ciprofloxacin; Esc = escitalopram; FX = fluoxetine; FZ = fluconazole; Am = amlodipine.

**Table S12. Calculated binding affinities and key interacting amino acid residues for ligands docked to Staphylococcus aureus MurB (PDB ID: 1HSK).**

| **Drug** | **Binding energy (kcal/mol)** | **Hydrogen bonds** | **Key interacting residues** |
| --- | --- | --- | --- |
| Ciprofloxacin | -9.1 | 6 | LEU197, ASN83, SER82, ASN80, SER143 |
| Fluoxetine | -8.6 | 4 | GLY79, VAL199, SER82 |
| Fluconazole | -8.4 | 3 | HIS196, SER143, SER115 |
| Escitalopram | -7.7 | 2 | SER235, TYR42 |
| Amlodipine | -6.8 | 3 | SER238, ARG188, GLY237 |

Binding energies were calculated using AutoDock Vina. Residue names are reported using standard three-letter amino-acid codes.

**Table S13. Calculated binding affinities and key interacting amino acid residues for ligands docked to Pseudomonas aeruginosa LasR (PDB ID: 3IX3).**

| **Drug** | **Binding energy (kcal/mol)** | **Hydrogen bonds** | **Key interacting residues** |
| --- | --- | --- | --- |
| Fluoxetine | -9.1 | 2 | SER235 and TYR42 |
| Ciprofloxacin | -8.8 | 3 | ARG61, TRP60, TYR93 |
| Fluconazole | -8.5 | 3 | ARG61, ASP65, LEU125 |
| Escitalopram | -6.8 | — | — |
| Amlodipine | -5.4 | 3 | GLY120, ASP43, ALA121 |

Binding energies were calculated using AutoDock Vina. LasR = quorum-sensing transcriptional regulator.

**Table S14. Calculated binding affinities and key interacting amino acid residues for ligands docked to Candida albicans lanosterol 14α-demethylase (CYP51; PDB ID: 5V5Z).**

| **Drug** | **Binding energy (kcal/mol)** | **Hydrogen bonds** | **Key interacting residues** |
| --- | --- | --- | --- |
| Fluconazole | -8.3 | 2 | ILE304 and THR311 |
| Ciprofloxacin | -8.3 | — | — |
| Fluoxetine | -8.2 | 1 | TYR505 (hydrogen bond); SER378 and MET508 (halogen contacts) |
| Escitalopram | -8.2 | — | — |
| Amlodipine | -6.8 | 4 | ILE471, CYS470, GLY472, TYR132 |

Binding energies were calculated using AutoDock Vina. CYP51 = lanosterol 14α-demethylase. Hydrogen-bond counts and other contacts are reported separately where applicable.
